# Parameter-free molecular super-structures quantification in single-molecule localization microscopy

**DOI:** 10.1083/jcb.202010003

**Published:** 2021-03-18

**Authors:** Mattia Marenda, Elena Lazarova, Sebastian van de Linde, Nick Gilbert, Davide Michieletto

**Affiliations:** 1 Medical Research Council Human Genetics Unit, Institute of Genetics and Molecular Medicine, University of Edinburgh, Edinburgh, UK; 2 Scottish Universities Physics Alliance, School of Physics and Astronomy, University of Edinburgh, Edinburgh, UK; 3 Scottish Universities Physics Alliance, Department of Physics, University of Strathclyde, Glasgow, UK

## Abstract

Understanding biological function requires the identification and characterization of complex patterns of molecules. Single-molecule localization microscopy (SMLM) can quantitatively measure molecular components and interactions at resolutions far beyond the diffraction limit, but this information is only useful if these patterns can be quantified and interpreted. We provide a new approach for the analysis of SMLM data that develops the concept of structures and super-structures formed by interconnected elements, such as smaller protein clusters. Using a formal framework and a parameter-free algorithm, (super-)structures formed from smaller components are found to be abundant in classes of nuclear proteins, such as heterogeneous nuclear ribonucleoprotein particles (hnRNPs), but are absent from ceramides located in the plasma membrane. We suggest that mesoscopic structures formed by interconnected protein clusters are common within the nucleus and have an important role in the organization and function of the genome. Our algorithm, SuperStructure, can be used to analyze and explore complex SMLM data and extract functionally relevant information.

## Teaser

Marenda et al. introduce a parameter-free algorithm to quantify super-structures and connected clusters in SMLM datasets. The algorithm is tested on simulated and experimental datasets, demonstrating that it can be used as an unbiased tool to extract information beyond simple clustering.

## Introduction

Single-molecule localization microscopy (SMLM; van de Linde et al., 2011; Schermelleh et al., 2010; Henriques et al., 2011; Sauer and Heilemann, 2017) is now commonly employed for quantitative analysis of molecular structures and interactions in both cell-based (Cisse et al., 2013; Kapanidis et al., 2018; Chong et al., 2018) and in vitro experiments (Revyakin et al., 2006; Deniz et al., 2008). Unlike other light microscopy techniques, SMLM achieves resolutions far beyond the diffraction limit, and its typical output is a list of 3D coordinates (or localization events) that are naturally analyzed using efficient clustering algorithms borrowed from quantitative big-data analysis and even astronomy (Owen et al., 2010; Sengupta et al., 2011; Garcia-Parajo et al., 2014; Baumgart et al., 2016; Spahn et al., 2016; Griffié et al., 2016). However, traditional clustering algorithms rely on user-defined parameters that are intrinsically intertwined with the notion of similarity that is necessary to define a cluster. These parameters can be either hypothesized by physical intuition or inferred via preemptive analysis (Burgert et al., 2017; Williamson et al., 2020; Malkusch and Heilemann, 2016), yet their choice has a significant impact on the results, in turn hindering the portability of clustering algorithms and the comparison between different datasets.

At the same time, recent evidence suggest that assemblies of proteins (Brangwynne et al., 2015; Larson et al., 2017; Strom et al., 2017; Sabari et al., 2018; Cho et al., 2018; Maharana et al., 2018; Chong et al., 2018) and chromatin (Bintu et al., 2018; Boettiger et al., 2016; Frank and Rippe, 2020) form functional complex structures that are not fully captured by standard clustering algorithms. For example, the heterogeneous nuclear ribonucleoprotein U (hnRNP-U), also called scaffold attachment factor A (SAF-A), is suggested to form a dynamic and functional mesh-like structure while interacting with RNA to maintain transcriptionally active genomic loci in a decompacted configuration (Nozawa et al., 2017; Michieletto and Gilbert, 2019). Other examples include SC35, a nuclear protein involved in RNA splicing and chromatin elongation (Lin et al., 2008) that displays localized nuclear speckles (Xie et al., 2006; Jackson et al., 2000), or actin and microtubules, which form elongated and interconnected networks involved in cell motility and division, as well as in the synaptic plasticity of dendritic spines (Resch et al., 2002; Rogers et al., 2003; Izeddin et al., 2011). Additionally, recent super-resolution studies indicate that chromatin is also functionally organized in connected nano-scale compartments (Prakash et al., 2015; Szabo et al., 2018; Nir et al., 2018; Maiser et al., 2020). Rapidly evolving methods of chromatin tracing (Boettiger et al., 2016; Wang et al., 2016; Beliveau et al., 2015; Nir et al., 2018; Bintu et al., 2018) and super-resolved imaging of the accessible genome (Xie et al., 2020) require sophisticated algorithms to analyze the topology of the generated paths (Goundaroulis et al., 2020). To understand the relationship between these complex structures and the underlying biological mechanism and functions of the genome (Bronshtein et al., 2015; Khanna et al., 2019; Leidescher et al., 2020 *Preprint*; Smeets et al., 2014), a more sophisticated and standardized analysis of SMLM data is urgently required.

It is clear that quantification of complex structures is a ubiquitous problem in molecular and cell biology, and it is intimately connected to cellular function. Motivated by this problem, here, we introduce a new algorithm termed SuperStructure, which extends in a novel and original way the popular density-based clustering algorithm DBSCAN. SuperStructure allows (1) a parameter-free detection and quantification of complex structures made of connected clusters in SMLM data and (2) a parameter-free quantification of the density of molecules within clusters.

Here, we demonstrate the capabilities of SuperStructure on simulated datasets and then use it to analyze two groups of experimental datasets: (1) nuclear proteins involved in RNA processing, namely SAF-A, hnRNP-C, and SC35; and (2) ceramide lipids involved in cellular trafficking at the membrane. We find that interconnections between clusters are abundant in classes of proteins in the hnRNP family and that they are surprisingly absent from ceramides, suggesting this feature is relevant for the biological function of SAF-A and hnRNP-C. Therefore, SuperStructure enables us to discover new facets of protein organization in human cells and provides a better understanding of the molecular mechanisms underlying the organization of subcellular (super-)structures.

Finally, since SuperStructure is parameter-free, it provides the community with a standardized tool for the discovery and quantification of complex patterns in SMLM data. Furthermore, beyond helping our understanding of complex biological structures, it might be used to assess the fluorophore blinking quality and thus offers versatility in assessing also technical imaging properties (van de Linde and Sauer, 2014; Hennig et al., 2015; Siegberg and Herten, 2011).

## Results

### SuperStructure algorithm

**Figure 1.**
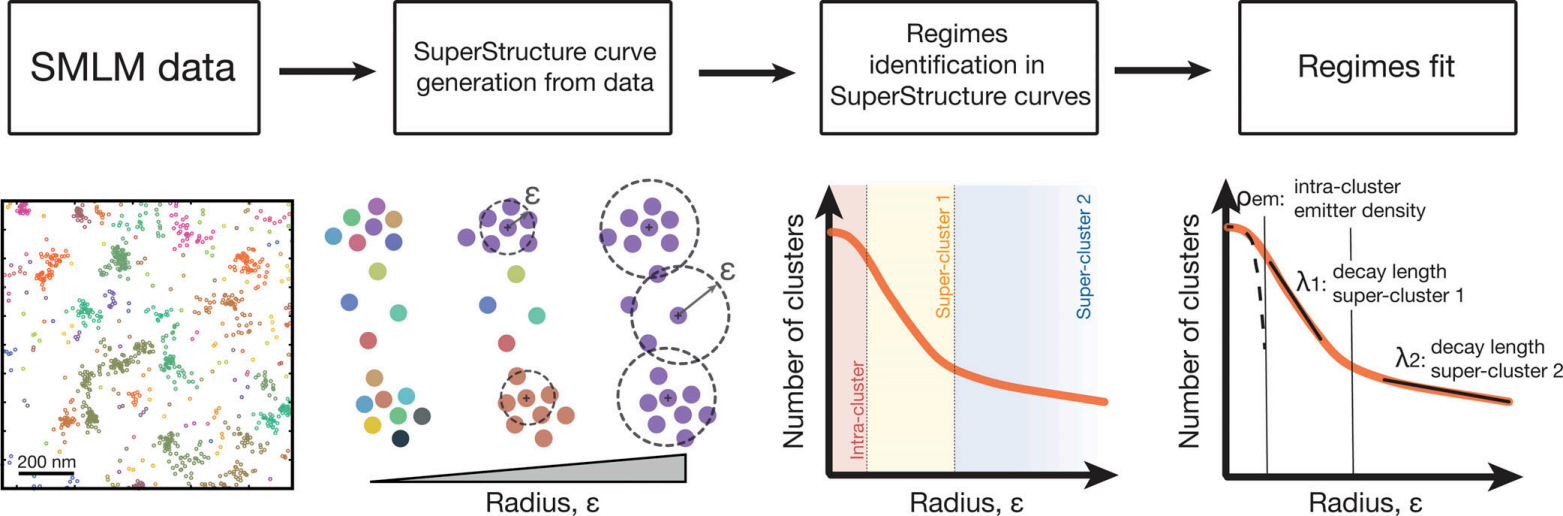
**Working principle of SuperStructure analysis.** Left: SMLM data are taken as input for the analysis. Center left: Cluster analysis is run using the DBSCAN algorithm with $N_{min}=0$ and ε progressively increasing in an adequate range for the system. SuperStructure curves describing the number of detected clusters $N_{c}$ as a function of ε are generated. Center right: SuperStructure curves are plotted and inspected to identify super-cluster regimes representing the onset of connected structures. Right: Intra- and super-cluster regimes are fitted with our models (see Materials and methods) to quantify the emitter density inside clusters $\rho_{em}$ and the connectivity among clusters (via the decay length $\lambda_{i}$ for super-cluster regime i).

SuperStructure is best explained in relation to the well-known DBSCAN algorithm. DBSCAN detects clusters by grouping together high-density localizations and classifies as outliers low-density ones (Ester et al., 1996). In practice, DBSCAN determines that a localization is part of a cluster if more than $N_{min}\;$ other localizations are found within a neighborhood distance ε (or if it is part of the neighborhood of another localization with this property). Conversely, SuperStructure extracts connectivity information from the rate at which the number of detected clusters $N_{c}$ changes with the neighborhood radius ε for a fixed $N_{min}$ (see Fig. 1). Indeed, the curves $N_{c}\left(\varepsilon \right)$ contain important overlooked information about the structure of connections. To simplify the analysis, and without loss of generality, we set $N_{min}=0,$ which means that we do not require a minimum number of localizations within the neighborhood to define a cluster. As a consequence, $N_{c}\left(\varepsilon \right)$ is necessarily a monotonically decreasing function, as for ε=0, every localization is detected as a single cluster and increasing ε yields fewer but larger clusters. Following on, the rate at which $N_{c}$ decays with ε is an indicator of how quickly localizations, and then clusters of localizations, coalesce, thus indicating how much localizations and clusters are connected.

**Figure S1.**
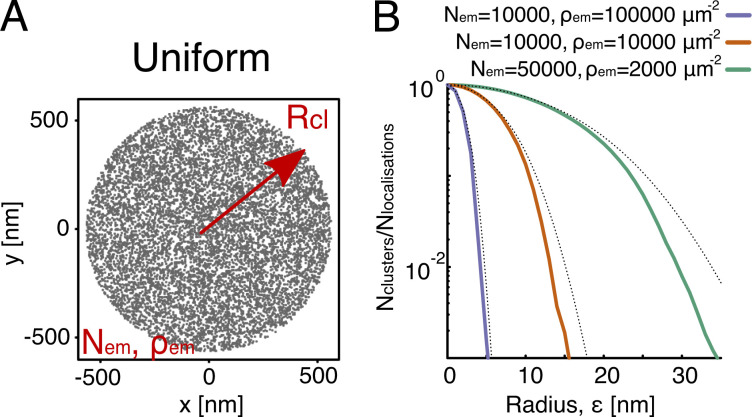
**The Poissonian functional form in the intra-cluster regime.** **(A)** To test the Poissonian functional form (Eq. 1) of the intra-cluster regime of SuperStructure curves, we simulated localizations inside clusters as a uniform distribution of $N_{em}$ points distributed within a circle of radius $R_{cl}.$ The resulting average density is $\rho_{em}.$ The number of points included in any circular subregion of radius ε is, on average, $n\left(\varepsilon \right)=\pi \rho_{em}\varepsilon^{2},$ and is in fact itself Poisson distributed. **(B)** To check the theoretical prediction of Eq. 1, we have created simulated datasets for various $\rho_{em}$ and $N_{em}.$ The theoretical predictions (dotted lines) with m=2 are in good agreement with the SuperStructure curves, indicating that indeed Eq. 1 correctly captures the behavior of uniformly distributed points forming one idealized cluster. However, note that for m=2, there is already an overcounting of clusters at large values of ε due to the fact that DBSCAN merges indirectly related emitters in a single big cluster. This suggests not to extend the summation to higher values of m. From Eq. 1, the end of the intra-cluster regime can be approximated by the width of the Poisson function, i.e., $\varepsilon^{*}≃3\kappa_{0}$ (at 99% confidence level), where $\kappa_{0}=1/\sqrt{\pi \rho_{em}}$ is the decay length identified by Eq. 1. This is confirmed by observing that predicted $\varepsilon^{*}$ for the curves are $\varepsilon^{*}\left(\rho_{em}=2,000\;\mu m^{-2}\right)≃38\;\;nm,$ $\varepsilon^{*}\left(\rho_{em}=10,000\;\;\mu m^{-2}\right)≃18\;\;nm,$ and $\varepsilon^{*}\left(\rho_{em}=100,000\;\mu m^{-2}\right)≃5.3\;\;nm,$ which correspond to $N_{cl}/N_{em}≃10^{-3}$ (when most of the points have been merged in a single cluster).

The $N_{c}\left(\varepsilon \right)$ curves provided by SuperStructure identify different clustering regimes (Fig. 1). The first (small ε) regime describes the merging of localizations within clusters (intra-cluster regime), the second (intermediate ε) regime captures the growth of clusters into super-structures (first super-cluster regime), and the third (large ε) regime describes the merging of super-clusters into higher-order super-structures (second/third super-cluster regimes). The $N_{c}\left(\varepsilon \right)$ curve in the first regime typically follows a Poissonian function (Eq. 1), and its decay rate is related to the density of emitters $\rho_{em}\;$ within the clusters (see Materials and methods and Figs. 1 and S1). The width of the Poisson function also sets the critical value of ε at which this first regime is expected to end (Eq. 2). On the other hand, the decay in the second and third regimes follows an exponential decay with characteristic length-scale λ and are highly dependent on the connectivity between (super-)clusters, as well as on the density of (super-)clusters (Eq. 4).

The number of super-cluster regimes depends on the homogeneity of both cluster distribution and connections. In the two extreme cases of a completely connected or unconnected homogeneous distribution of clusters, we expect a single super-cluster regime. However, while in the former case this regime is exponential (because the clusters are connected), in the latter it assumes a Poissonian functional form (see respectively Eqs. 4 and 3). This is not surprising, as free (unconnected) clusters that are randomly distributed behave (on a larger scale) as single emitters inside clusters (see Materials and methods and Fig. S1). Also, in the case of clusters embedded in a random distribution of other localizations (such as noise), we obtain a Poissonian decay. Importantly, a random distribution of localizations (also at high density) is different from “connected” clusters, where nearby localizations are mostly distributed in between clusters. As a result, the curves generated by SuperStructure allow us to identify the presence/absence of connectivity by investigating the functional form of the curves, as well as to extract their decay rates.

In heterogeneous systems that display a mix of randomly dispersed localizations/clusters and connected ones over similar length-scales, we strongly recommend restricting the analysis with regions of interest (ROIs) over subregions that display qualitatively similar phenotypes. A good example of heterogeneous system is given by the nuclear protein SC35, which we analyze below. Restricting the analysis to ROIs is also recommended when quantifying nuclear or cellular substructures that display boundaries. Masking localizations falling outside these boundaries allows SuperStructure to generate cleaner curves that are easier to interpret.

To quantify the intra-cluster density and (super-)cluster connectivities, one needs to define boundaries between regimes and to fit every regime with the corresponding function (see Eqs. 1, 3, and 4). Regime boundaries and fitting ranges can be selected either manually (where curves change their decay properties) or by rigorously running a preemptive goodness-of-fit test. For instance, once the rough regime range has been identified and fitted, one can modify the fit window to identify the boundaries of the regime outside which the fit is no longer acceptable. Arguably, the optimum regime is found by identifying the best goodness-of-fit window (e.g., the range with the minimum χ^2^). It is also possible to define a single function fitting the entire curve by (1) defining a piecewise function where every “piece” is the fit of the corresponding regime or (2) adding together the contribution of the different regimes (appropriately weighted).

The workflow for the application of SuperStructure is shown in Fig. 1 and is described in detail in Materials and methods. Additionally, the codes and scripts are open source and available at git repository (see below).

### Characterizing SuperStructure feature extraction from simulated SMLM data

**Figure 2.**
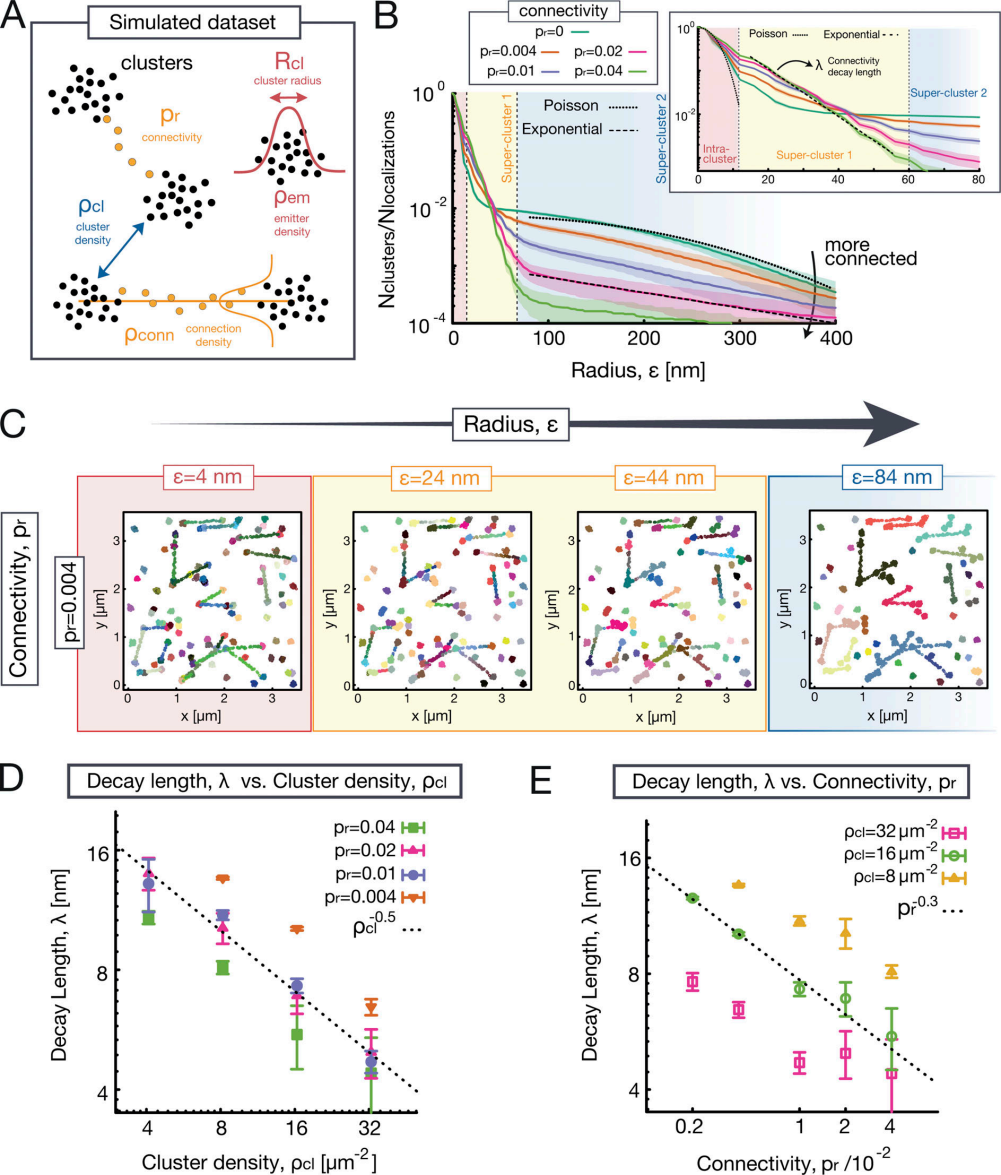
**Evaluating SuperStructure on simulated datasets.** **(A)** Sketch representing the artificial dataset consisting of interconnected clusters of localizations on a 2D plane. Clusters are characterized by an internal density of localizations $\rho_{em}$ and radius $R_{cl}$ and are randomly distributed on the plane at an average cluster density $\rho_{cl}.\;$ Clusters can be connected by a sparse point distribution with probability $p_{r},$ and connections have a density of points $\rho_{conn}$ (controlled by the $p_{r}{}_{conn}$ parameter). **(B)** Average SuperStructure curves (zoomed in the inset) for simulated datasets with different connectivity $p_{r}.$ Other parameters are kept fixed: average cluster radius $R_{cl}≃40\;\;nm,$ emitter density within clusters $\rho_{em}=16,000\;\mu m^{-2},$ cluster density $\rho_{cl}=8.2\;\;\mu m^{-2},$ and $p_{r}{}_{conn}=0.5$ (which fixes the density of emitters within connections $\rho_{conn}).$ The curves show the number of detected clusters normalized by the total number of localizations. Curves are the average of 20 independent simulated datasets. Shaded regions represent the standard deviation from the average. Three regimes can be distinguished: (1) the intra-cluster (red), (2) the first super-cluster (yellow), and (3) the second super-cluster (blue). The decay in the intra-cluster regime corresponds to a Poisson avoidance function with density parameter $\rho_{em}=16,000\;\mu m^{-2}$ (Eq. 1, dotted line in the inset). The first super-cluster regime can be fitted by a single exponential (Eq. 4, dashed line in the inset) which returns an effective decay length λ. The second super-cluster regime can be fitted with another exponential if $p_{r}\neq 0$ (Eq. 4, dashed line in the main figure). In case of $p_{r}=0,$ there is only one super-cluster regime, and it follows a Poisson function with density parameter $\rho_{cl}=8.2\;\mu m^{-2}\;$ (Eq. 3, dotted line in the main figure). **(C)** Snapshots of detected clusters for an artificial dataset with connectivity $p_{r}=0.004$ and by progressively increasing the value of the radius ε=4, 24, 44, 84 nm. **(D)** Decay length λ versus cluster density $\rho_{cl}$ scales as $\rho_{cl}^{-0.5}$ for any value of connectivity $p_{r}.$ **(E)** Decay length λ versus connectivity $p_{r}$ scales as $p_{r}^{-0.3}$ for different values of $\rho_{cl}.$ In D and E, 20 independent datasets were fitted with Eq. 4, and the resulting λ values were averaged. Vertical bars represent the standard deviation from the average.

To evaluate the performance of SuperStructure, we analyzed artificial datasets consisting of interconnected clusters of localizations on a 2D plane (see Fig. 2 A). Clusters are homogeneously and randomly positioned on the plane with a cluster density $\rho_{cl}\;=\;8.2\;\mu m^{-2}$ that is comparable to that of some nuclear proteins (see below). Every cluster has average radius $R_{cl}∼\;40\;nm$ and an overall internal localization density $\rho_{em}=N_{em}/\pi R_{cl}^{2}=16,000\;\mu m^{-2},$ where $N_{em}$ is the number of localizations per cluster. Pairs of clusters are connected with probability $p_{r}$ by a sparse point distribution and only if the distance between the clusters is less than b=1 μm. These choices allow us to readily tune the degree of “connectivity” in the system by varying a single parameter $p_{r}.$ A second parameter, $p_{r_{conn}},$ is introduced to control the density of localizations within the connections $\rho_{conn}$ (see Materials and methods for details).

**Figure 3.**
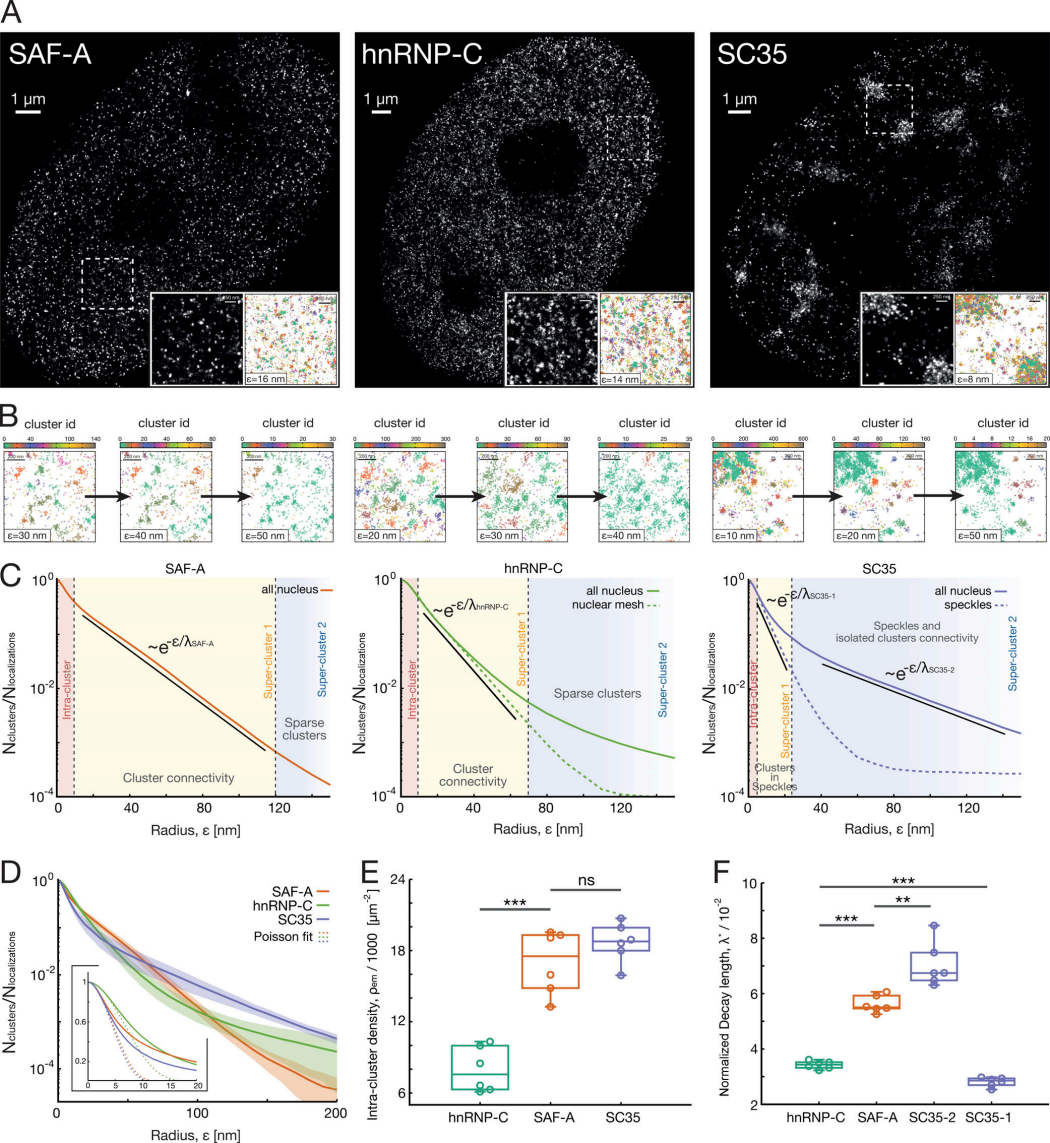
**Application of SuperStructure algorithm to SAF-A, hnRNP-C, and SC35 SMLM data.** **(A)** Reconstructed dSTORM images by using the shifted histograms method with a pixel size of 10.6  nm. Insets of $4-\mu m^{2}$ size of reconstructed dSTORM images and spatial positions of the data. Palettes represent the cluster ID computed by running SuperStructure with $N_{min}=0$ and ε  at the start of the first super-cluster regime. **(B)** Identified clusters for increasing values of ε  in the regimes where clusters merge. **(C)** Normalized average SuperStructure curves in the range [0:150]  nm. The number of detected clusters has been normalized with the total number of localizations in the system. The average is calculated over six independent datasets (nuclei). Solid curves indicate that SuperStructure analysis was run on the entire nucleus, and the resulting curves for the six independent datasets were averaged (all-nucleus curves). Dashed curves indicate that SuperStructure analysis was run in five local ROIs for each of the six nuclei, and then the curves of each region (for each nucleus) were averaged (local curves). In hnRNP-C, these local regions were chosen within the nuclear mesh (to exclude nucleoli), and in SC35, they were chosen within speckles. Vertical dashed lines highlight different SuperStructure regimes: intra-cluster, first super-cluster, and second super-cluster regime. For SAF-A and hnRNP-C, the exponential regime of clusters merging (first super-cluster regime) is highlighted with a solid straight line. In case of SC35, two regimes are highlighted, the merging of clusters within speckles (first super-cluster regime) and the merging of speckles with isolated clusters (second super-cluster regime). **(D)** Normalized all-nucleus average SuperStructure curves in the range [0:200] nm for the three proteins. Average is computed over six nuclei. Shaded regions represent standard deviation from the average. Poisson fits (Eq. 1) for the intra-cluster regime at small ε are shown in the inset. **(E)** Intra-cluster density of emitters $\rho_{em}$ as parameter of Poisson fit for six independent nuclei (Eq. 1). **(F)** Normalized decay length $\lambda^{*}$ for the super-cluster regimes highlighted in C for six independent nuclei. SuperStructure curves were fit with Eq. 4 to extract the decay length λ, and then the normalization $\lambda^{*}=\lambda /\rho_{cl}^{-1/2}$ was performed (where $\rho_{cl}$ is the detected cluster density at the beginning of each regime of interest). Details are explained in Materials and methods and Fig. S3. P values were calculated using a Student’s *t* test: ns, P > 0.05; **, P < 0.01; ***, P < 0.001.

The length-scales associated to density of emitters inside clusters $\rho_{em}$ and inside connections $\rho_{conn}$ define the boundaries among the three regimes of $N_{c}\left(\varepsilon \right)$ (Fig. 2 B): (1) for ε≲12 nm, the intra-cluster regime follows a Poissonian decay (Eq. 1) with density parameter $\rho_{em}=16,000\mu m^{-2}$ (as expected, since it was set by construction); (2) for intermediate values of ε, the exponential super-cluster regime dominates (Eq. 4), and the fusion of connected clusters takes place (see inset of Fig. 2 B); (3) for ε≳60 nm, we expect to observe the coalescence of super- and nonconnected clusters in a second super-cluster regime; this is captured by a second exponential for $p_{r}\neq 0$ (Eq. 4). Conversely, for $p_{r}=0,$ we observe a single super-cluster regime that is well fitted by a Poissonian function with lower density (Eq. 3), as it corresponds to the density of clusters rather than emitters within clusters (see dark green curve in Fig. 2 B).

**Figure S2.**
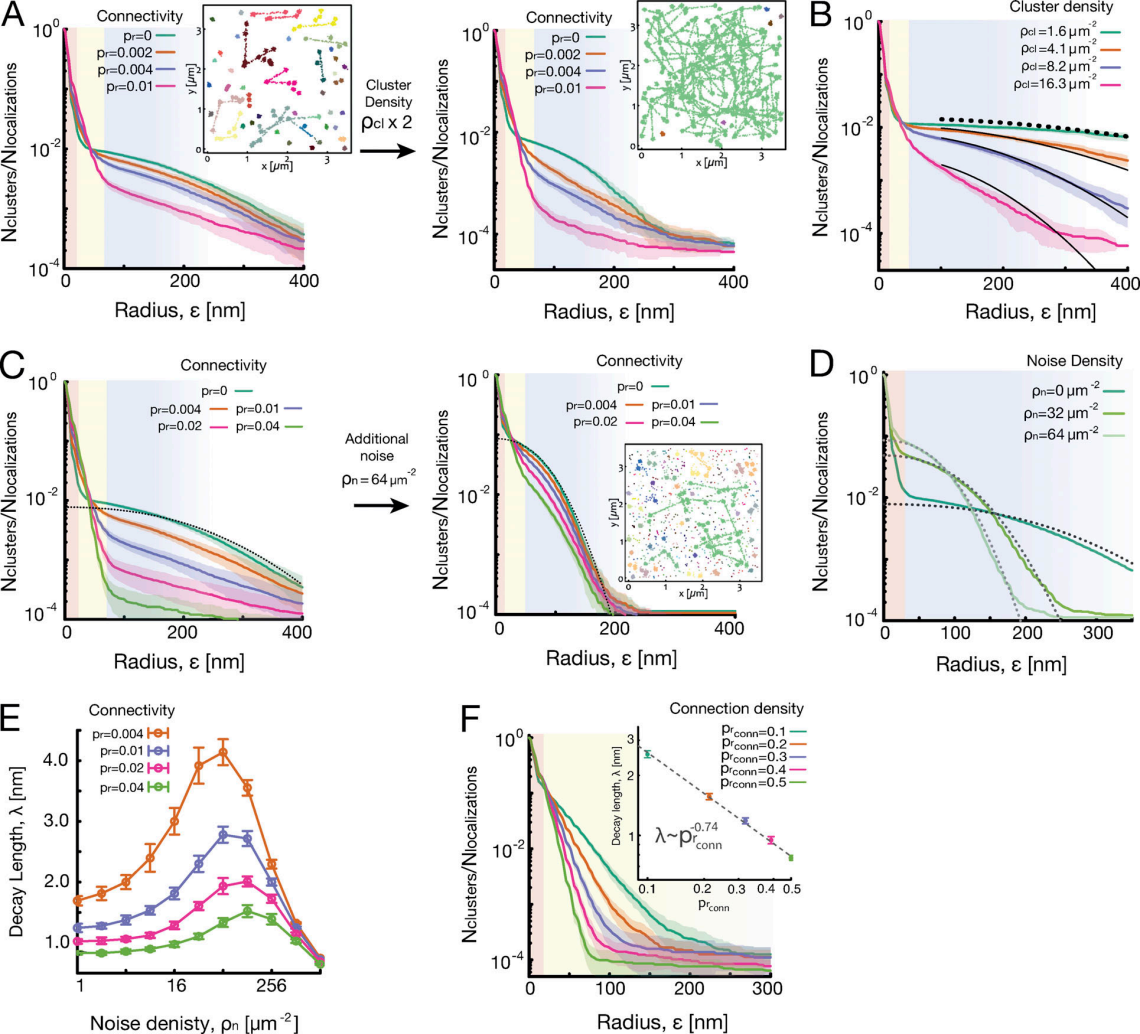
**Average SuperStructure curves for simulated datasets in different conditions.** SuperStructure analysis was run on 20 independent datasets (each in the same condition), and the resulting curves were then averaged. Shaded regions represent the standard deviation from the average. Parameters are set to their standard values if not otherwise specified (see Materials and methods). Palettes in the inset configurations represent cluster analysis at ε=80  nm. **(A)** Locally connected clusters with different grades of connectivity and doubling the cluster density (from left to right): $\rho_{cl}=8.2\;\mu m^{-2}$ (left) and $\rho_{cl}=16.3\;\mu m^{-2}$ (right), connection density $p_{r}{}_{conn}=0.5,$ and no noise and different values of connectivity $p_{r}.$ The higher cluster density makes SuperStructure curves more markedly distinct as a function of $p_{r}$ compared with the same curves for a lower density. **(B)** Locally connected clusters with low connectivity and increasing cluster density: connectivity $p_{r}=0.002,$ connection density $p_{r}{}_{conn}=0.5,$ and no noise and different cluster densities $\rho_{cl}.$ The first super-cluster regime maintains the single exponential decay, but the decay length λ decreases with the cluster density. In the main text, we showed that this dependence goes as $\lambda \propto \rho_{cl}^{-1/2}.$ Also, the exponential decay $\lambda_{2}$ of the second super-cluster regime decreases with the density of clusters, and this regime evolves from a Poisson-like (low $\rho_{cl})$ to an exponential decay (high $\rho_{cl}).$ This behavior seems to be a pure effect of the cluster density, as all other parameters remain unchanged. Black curves are Poisson decays attempts $∼e^{-\pi \rho \varepsilon^{2}}$ to fit the second super-cluster regime. **(C)** Locally connected clusters with different grades of connectivity and sparse noise addition: cluster density $\rho_{cl}=8.2\;\mu m^{-2},$ connection density $p_{r}{}_{conn}=0.5,$ noise density $\rho_{n}=0\;\mu m^{-2}$ (left)/$\rho_{n}=64\;\mu m^{-2}$ (right), and different values of connectivity $p_{r}.$ With high noise (eight times the cluster density), the second super-cluster regime becomes Poissonian; the first super-cluster regime maintains its typical exponential decay, but the decay length is altered. Dotted lines represent fit with Eq. 3 for ε∈[70:300] nm. **(D)** Unconnected clusters of points with increasing density of noise (other parameters are the same as C). Eq. 3 well describes the decay of the curves in the intercluster regime, with the density parameter $\rho_{cl}$ and $\rho_{cl}+\rho_{n},$ respectively, in absence and presence of noise. **(E)** Average decay length of the first super-cluster regime for the connected systems represented in C as function of noise density $\rho_{n}.$ The fit to calculate the decay length λ  has been made for ε∈[20,60] nm for 20 independent datasets. Values of λ are then averaged. Bars represent the standard deviation from the average. Decay lengths for systems with different connectivities $p_{r}$ are distinguishable as long as the noise density is below the connection density (~500 μm^−2^). However, low noise density also alters the estimation of the decay length. The alteration is less severe for highly connected clusters. **(F)** Fully connected meshes of clusters with increasing density of the mesh: cluster density $\rho_{cl}=\;8.2\;\mu m^{-2},$ connectivity p=0.025, and no noise and different values of connection density $p_{r_{conn}}.$ The super-cluster regime is unique, the decay is exponential, and the decay length λ  decreases with the density of the mesh. Fit of λ was performed for ε∈[20:60] nm. The inset shows the dependence of λ on $p_{r_{conn}}$ in a fully connected mesh, which is $\lambda ∼p_{r_{conn}}^{-0.74}.$

Examination of Fig. 2 B (inset) highlights the exponential behavior of the super-cluster regime (2) for different values of connectivity $p_{r}.$ Importantly, a larger $p_{r}$ results in an effectively shorter decay length (or larger spatial rate of merging) for the regime in which clusters merge into super-clusters. This strongly suggests that the effective decay length (or rate) mirrors the connectedness of the underlying super-structures (Fig. 2 C). In fact, these simulations reveal that the decay length represents the combined contribution of cluster density $\rho_{cl}\;$ and connectivity $p_{r}.$ A larger density of clusters can impact the decay length as much as a larger connectivity, as shown by simulations at fixed $p_{r}$ and different $\rho_{cl}$ (Fig. 2 D; and Fig. S2, A and B). In particular, we find that the functional form of the decay length is $\lambda ∼\rho_{cl}^{-1/2}\;p_{r}^{-0.3}$ (Fig. 2, D and E). The cluster density contribution is $∼\rho_{cl}^{-1/2},$ as it depends on the typical distance between clusters and is relevant when comparing datasets with different cluster density. By combining SuperStructure with a cluster analysis, one can estimate $\rho_{cl}$ and normalize λ to obtain the pure connectivity contribution in the decay length: $\lambda^{*}=\lambda /\rho_{cl}^{-1/2}.$

Finally, in order to characterize the contribution to the $N_{c}\left(\varepsilon \right)$ curves coming from the density of localizations within the connections, we further simulated SMLM datasets with a fixed, large connectivity $p_{r}$ and varied the density of points in the connections by tuning $p_{r}{}_{conn}$ (see simulated datasets in Figs. 2 A and S2 F). As expected, we observe a single super-cluster regime, and the denser the connections, the shorter the decay length. This indicates that our algorithm is able to describe not only how well clusters are connected (i.e., the number of connections per cluster) but also how strongly they are connected (i.e., how dense the connections are). These features are likely to be highly relevant for nuclear proteins.

Before applying this methodology to experimental data, we also tested the effect of random noise in the system (i.e., unconnected isolated localizations from biological or technical sources). We observed that in presence of random noise the decay of SuperStructure curves becomes Poissonian for large ε (see Fig. S2 C) with an effective density ρ larger than the cluster density (see Fig. S2 D). Decay lengths in the first super-cluster regime (yellow regime) are still distinguishable even in presence of noise at reasonable density (albeit smaller than the connection density), but their absolute values are altered, with weakly connected systems more severely affected (see Fig. S2 E). These observations suggest that, as in most analysis algorithms, large noise might obscure exponential decays of connected systems. In case a single Poissonian behavior or a combination of exponential and Poissonian decay is found in the SMLM dataset, it is therefore important to combine SuperStructure with an independent cluster analysis at different length scales (e.g., at three or four selected values of ε) and a direct observation of the dataset in order to exclude the presence of hidden connectivity.

### Quantification of super-structures in nuclear proteins

We now examine biological data and apply SuperStructure to dSTORM data acquired for three different nuclear proteins (Fig. 3, A and B): the serine/arginine-rich splicing factor SC35, hnRNP-C, and hnRNP-U (also known as SAF-A). These proteins are abundantly expressed in the nucleus of human cells and are involved with RNA processing at different stages. SC35 is necessary for RNA splicing, while hnRNPs are implicated not only in the regulation and maturation of mRNA but also in chromatin structure (Nozawa et al., 2017; Xiao et al., 2012; Caudron-Herger et al., 2011). In particular, SAF-A is thought to form a dynamic homogeneous mesh that regulates large-scale chromatin organization by keeping gene-rich loci in a decompacted state (Nozawa et al., 2017; Michieletto and Gilbert, 2019). Hence, capturing the organization of this protein beyond the traditional single-cluster analysis is an important step toward understanding how it regulates chromatin structure in different cell stages and conditions.

Curves obtained from SuperStructure analysis after masking signal in the nuclear region are shown in Fig. 3 C, where we highlighted the super-cluster regimes discussed above. Global nuclear analysis is represented by filled curves, while analysis on localized ROIs is represented by dashed ones (hnRNP-C nuclear mesh and SC35 speckles). Both hnRNPs display a first super-cluster regime for which the curves decay as exponentials, suggesting that within this range, distinct clusters are in reality connected. Interestingly, while SAF-A displays a unique long super-cluster regime, hnRNP-C seems to also show a second exponential regime (filled curve). However, this regime appears at very large values of ε and is due to sparse clusters of localizations in nucleoli. Running SuperStructure on ROIs with nucleoli masked out (dashed line) indeed generates a single exponential function, confirming that hnRNP-C clusters are fully connected. We can therefore conclude that both hnRNPs exhibit a single exponential regime, typical of fully connected meshes. On the other hand, SC35 displays exponentials with different characteristic decay rates in two distinct and significant super-cluster regimes (filled curve), one for intermediate ε∈ [10,20] nm, when clusters inside speckles merge (first super-cluster regime), and another one for large ε∈ [40,150] nm, indicating that speckles merge together and with isolated clusters (second super-cluster regime). The SC35 connectivity is further confirmed by running SuperStructure on ROIs masking the speckles, as we observed a clear single exponential decay (dashed line). These regimes are further confirmed by directly looking at the arrangement of identified clusters for certain values of ε (see Fig. 3, A [inset] and B).

**Figure S3.**
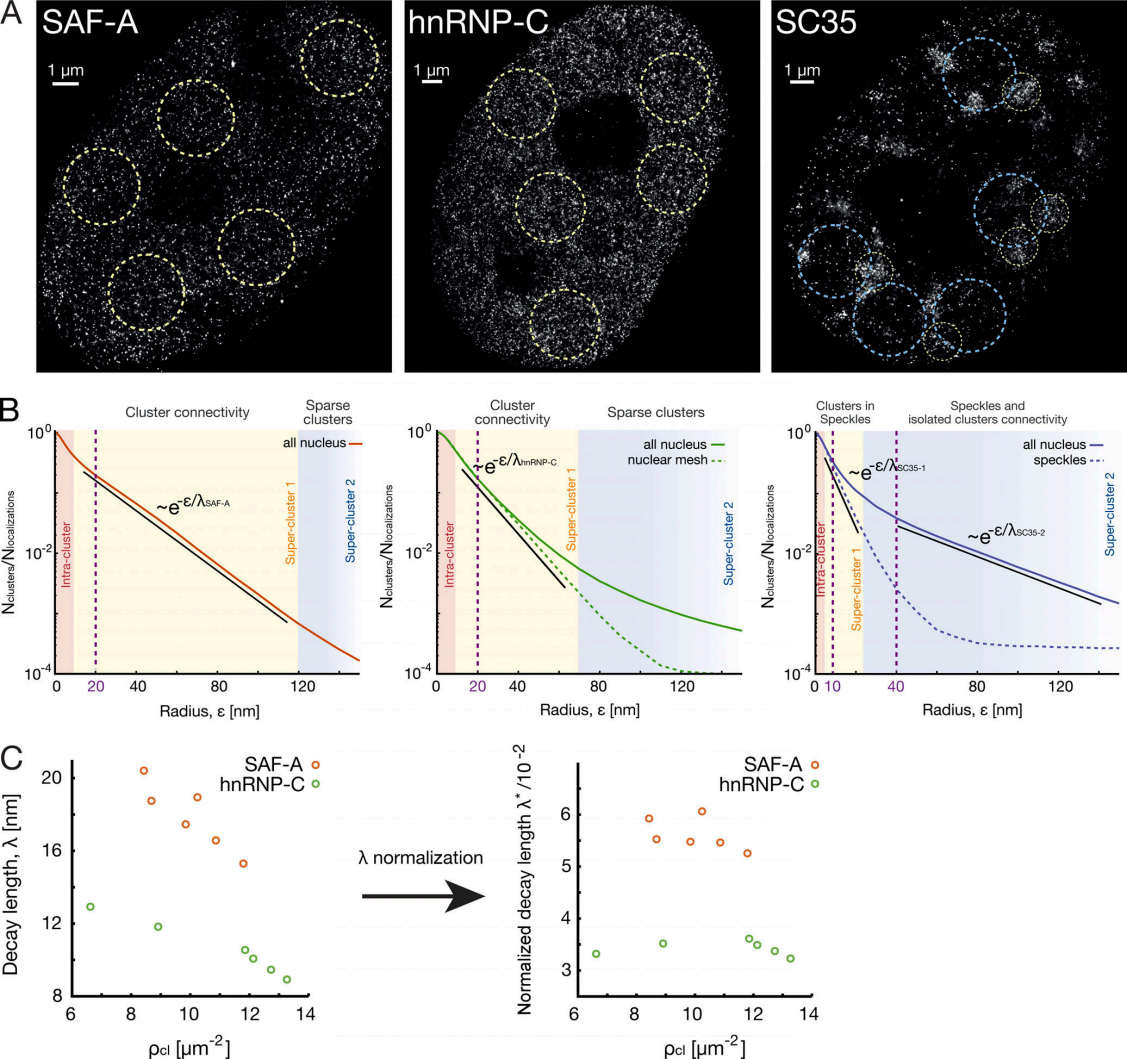
**Details on **λ **normalisation and proof that connections are not technical artifacts in nuclear protein data.** **(A)** dSTORM reconstructed images of SAF-A, hnRNP-C, and SC35 in a single cell where local circular regions for cluster density estimation purpose are highlighted. In the case of SC35, two different region types are used, one inside speckles for the first exponential regime and one outside speckles for the second exponential regime. In the case of hnRNP-C and SC35, local circular regions were also used to compute SuperStructure local curves and the decay length λ in the first super-cluster regime, as explained in Materials and methods. **(B)** Average SuperStructure curves for SAF-A, hnRNP-C, and SC35 are shown and explained in the main text. Solid lines are the result of all-nucleus analysis, while dashed lines are the result of a local analysis (in local circular regions). Exponential regimes of interest are highlighted, as well as the values of ε at which the cluster analysis is made for cluster density estimation purposes (purple dashed vertical line). **(C)** Check that connections are not the result of technical artifacts due to bad blinking quality both in SAF-A and hnRNP-C data by monitoring λ (left) and $\lambda^{*}$ (right) for different cluster densities $\rho_{cl}.$ The bad blinking quality of fluorophores would lead to localization inaccuracy of emitters at the borders of protein clusters, and this in turn could lead to pseudo-connections between clusters. However, these pseudo-connections would be proportional to the cluster density; a higher cluster density would result in stronger pseudo-connections, which would reflect to a decrease of $\lambda^{*}$ with the cluster density. $\lambda ,\;\;\rho_{cl},$ and $\lambda^{*}$ were calculated for the six independent nuclei, as explained in Materials and methods, and are shown in Table S1. Every nucleus can be considered as a system where the blinking conditions are the same, but cluster densities may vary due to statistical fluctuations. While λ (left) decreases with $\rho_{cl},\;$ as expected, $\lambda^{*}$ (right) is constant for different densities, ruling out the hypothesis that connections are artifacts due to bad blinking quality.

From the SuperStructure curves, we first obtained the density of intra-cluster emitters by fitting the intra-cluster regime with the Poisson function (Eq. 1). Interestingly, both SAF-A and SC35 form clusters with similar densities, while hnRNP-C clusters are less dense (see Fig. 3, D and E). Then, in order to have a quantitative description of the clusters/speckles connectivities, we fitted the curves in the exponential regimes (Eq. 4) to extract the decay length λ. However, a direct comparison is possible only by normalizing decay lengths by the cluster/speckle density (see Materials and methods for details and Fig. S3, A and B). Fig. 3 F highlights that while hnRNP-C has a short normalized decay length $\lambda^{*}$ due to the highly connected clusters, SAF-A displays a weaker decay (larger $\lambda^{*})$ due to sparser connections. Finally, SC35 displays a first (intra-speckle) very connected regime, even more than that of hnRNPs (smaller $\lambda^{*}).$ This is followed by a second (inter-speckle) regime that shows a cluster connectivity weaker than that of hnRNPs.

In summary, our analysis revealed that while different nuclear proteins may have similar cluster sizes or densities of emitters within clusters (e.g., SAF-A and SC35), they have distinct super-cluster arrangements and connectivities. For instance, we find that the super-structures inside nuclear speckles are more connected than those formed by hnRNPs and also very dense (see Fig. 3, E and F; and Table S1). We stress that these features, which we further verified not emerging from technical artifacts (see Fig. S3 C), cannot be quantified using standard clustering algorithms or pair-correlation functions. Additionally, the analysis in Fig. 3, E and F shows that our method is sensitive enough to distinguish connectivity features of two closely related wild-type hnRNPs in cell-based experiments.

The results presented in Fig. 3 give us confidence not only that SuperStructure can be applied to a variety of nuclear wild-type or mutated proteins in different cells, cell stages, and conditions, but also that it has the capability to extract unique features that may yield new mechanistic insights into the functioning of such proteins. For instance, the analysis of SC35 reveals that speckles are themselves made of clusters that are as heavily interconnected as the clusters formed by hnRNP proteins. Given the fact that all these proteins interact with RNA, our findings suggest that RNA binding may facilitate the formation of connections between clusters of proteins; in turn, this also points to a suspected structural role of noncoding RNAs in structuring the organization of the nuclear interior (Hall and Lawrence, 2016). Studying the effect of RNA depletion on the super-cluster connectivity is therefore a natural next step to perform in the future.

In general, while certain mutations or conditions may not alter the size of protein cluster itself, they may affect the connectivity between clusters. In these cases, the analysis provided by SuperStructure would be invaluable and indeed essential to reveal the underlying mechanisms that guide the formation of such protein assemblies.

### Ceramide clusters at the plasma membrane are not connected

**Figure 4.**
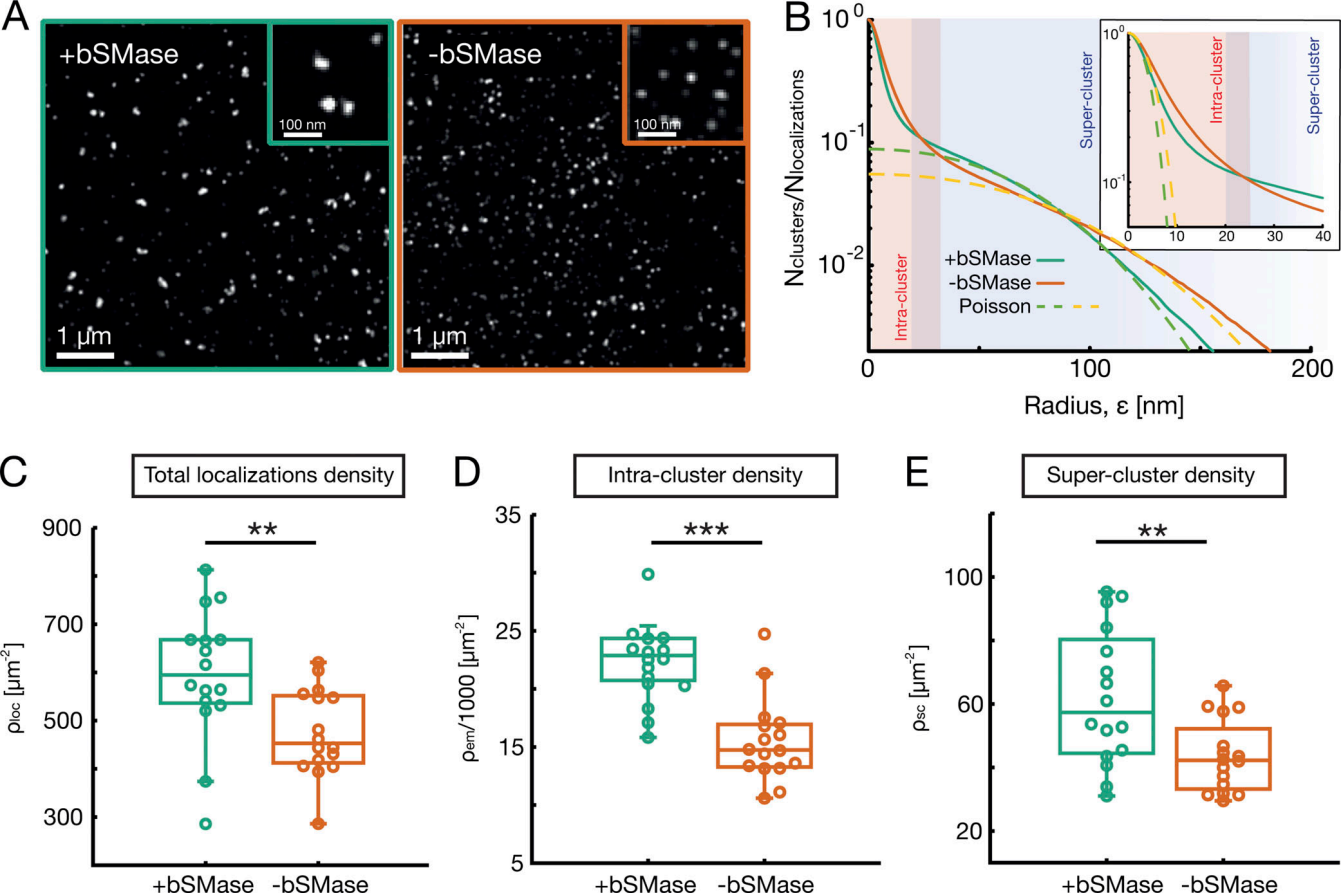
**Application of SuperStructure algorithm to ceramide data.** Analysis was performed on published data (Burgert et al., 2017). **(A)** dSTORM reconstruction of ceramides dataset using the shifted histogram method. The left panel represents signal from cells treated with bSMase; the right panel is a control without treatment. **(B)** SuperStructure curves of the two conditions for the entire dataset. Curves show the number of detected clusters normalized by the total number of localizations. The red region highlights the intra-cluster regime, while the blue region highlights the Poissonian unconnected super-cluster regime. The shaded purple region highlights the horizontal shift between the two curves. Dashed lines represent Poisson fits at low and high ε. **(C–E)** Average density of total localizations (C), intra-cluster density extracted as parameter from Poisson fit (Eq. 1; D), and overall density in the super-cluster regime extracted as parameter from Poisson fit (Eq. 3; E) for +bSMase and −bSMase treatment datasets. Calculations and fits were performed on data and SuperStructure curves from 16 independent circular regions of radius r=1.5 μm within the original dataset. P values were calculated using a Student’s *t* test: **, P < 0.01; ***, P < 0.001.

**Figure S4.**
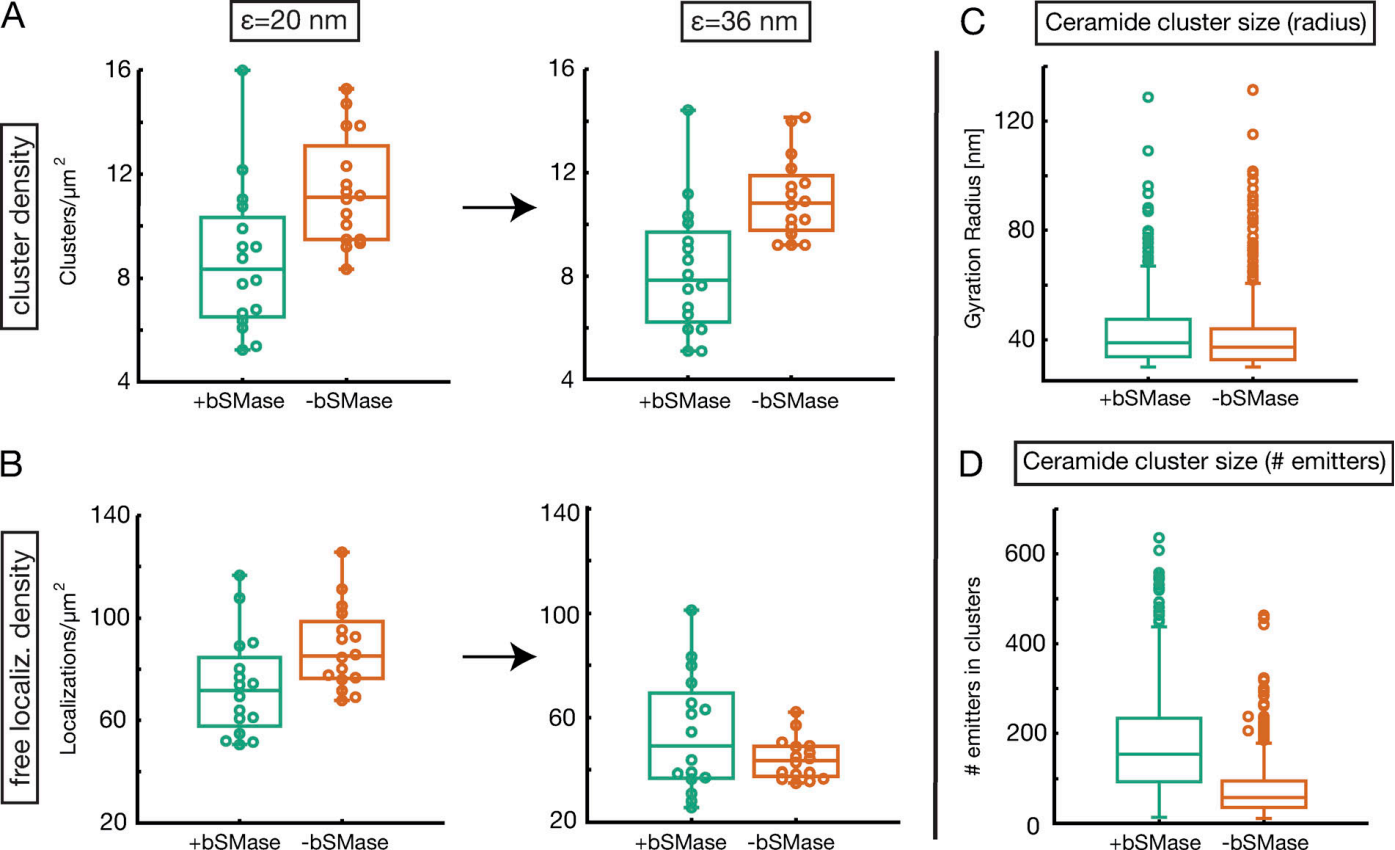
**Absence of local connectivity and confirmation of original paper results in ceramide data.** **(A and B)** The absence of local connectivity was confirmed by analyzing cluster density (A) and sparse localization density (B) in the crossover range. We monitored the density of ceramides clusters and that of free emitters at $\varepsilon_{1}=20\;\;nm$ and $\varepsilon_{2}=36\;nm.$ To calculate cluster density, DBSCAN was run at $N_{min}=0$ and at the given value of ε, and we kept only clusters with at least 10 particles. The remaining particles were considered as free localizations. Clusters and free localizations were detected at $N_{min}=0$ for 16 independent circular regions. The number of clusters remains constant in the considered ε regime, while the free localizations density significantly decreases (more severely for −bSMase cells). As a consequence, we can state that there is no significant merging of ceramide clusters, only embedding of nearby free localizations in already-formed clusters. **(C and D)** Confirmation of the original paper’s results by calculating the ceramide cluster size both as gyration radius (C) and number of emitters (D). Protein clusters were detected at $N_{min}=0$ at $\varepsilon^{+}=20\;nm$ and $\varepsilon^{-}=24\;nm.$ In accordance with the analysis in the paper, we looked at the size of clusters with a radius >30 nm. Note that +bSMase ceramide clusters consist of (on average) 180 emitters in a circle of radius 42 nm. The resulting density is 32,500 μm^−2^. This result is approximately in line with our prediction obtained with the Poisson intra-cluster fit by considering that the standard deviation of both cluster radius and emitters is high. Similarly, −bSMase clusters have on average 78 emitters in an average cluster radius of 40 nm. The resulting density is 15,500 μm^−2^.

To test our algorithm on a different class of molecules, we applied SuperStructure on published dSTORM datasets (Burgert et al., 2017) taken on ceramides-membrane lipids involved in cellular trafficking (Fig. 4 A). The authors (Burgert et al., 2017) found that bacillus cereus sphingomyelinase (bSMase) treatment increases the size of ceramides clusters and the overall localization density. By applying SuperStructure analysis (Fig. 4 B), we confirmed these results and further detected that the difference in localization density persists inside clusters (see Fig. 4, C and D; and Fig. S4, C and D). Furthermore, we detected the absence of connectivity between clusters, as the large ε regime is well captured by a Poisson function (Eq. 3) and not by an exponential (see Fig. 4, B and E). In other words, clusters of ceramides behave as unconnected, uniformly and randomly distributed emitters. The possibility of local connectivities at intermediate ε has also been ruled out, as no merging of clusters was directly observed (see Fig. S4, A and B). The crossing of the curves at  ε≃25 nm is a consequence of the difference in overall localization density (which in turn causes a horizontal shift between the curves; see Fig. 4, B [inset] and C), rather than a difference in local connectivities. The notable absence of connections between clusters of ceramides further supports that the ones detected in hnRNP-U/C and SC35 are significant.

### Limitations and potential interpretation pitfalls

While we have provided evidence that SuperStructure can detect connected clusters and distinguish them from noise (at low density) or unconnected but dense clusters, in this section, we discuss potential pitfalls and interpretation issues.

First, as mentioned earlier, datasets should always be segmented in order to identify the main ROI. Spurious localizations outside the ROI (e.g., outside of the nucleus, if we are interested in nuclear proteins) may affect the curves generated by SuperStructure and render their interpretation difficult. An analogous issue may arise if the localizations are embedded within heterogeneous structures, as in the case of SC35 proteins that form structures strongly connected within nuclear speckles and weakly connected outside speckles (see Fig. 3). Due to this mixed behavior over similar length-scales, it is recommended to restrict the analysis to regions that display similar structural phenotypes. Even better, and to be preferred when possible, is to label the region or structure of interest with orthogonal markers.

The key difference between connected and unconnected (albeit possibly more clustered) structures is the functional form of the SuperStructure curves. However, in some cases, Poisson curves may be difficult to distinguish from exponentials (especially over short intervals). In this case, the best way to identify connected clusters (and distinguish them from noisier or more clustered subregions) is to restrict the analysis over smaller ROIs to avoid potential contaminations and to perform goodness-of-fit tests on the curves. Additionally, in these complex cases we also suggest performing an independent cluster analysis over different length-scales and directly inspecting the results.

As with all computational algorithms, the danger of incorrect interpretation can be addressed with quality control. In the case of SuperStructure, this means directly monitoring the formation of connected clusters/structures while increasing ε. Nonetheless, thanks to its parameter-free execution, SuperStructure may currently offer one of the safest ways to analyze SMLM data.

## Discussion

In this work, we have introduced a novel algorithm that extends the traditional idea of cluster analysis of SMLM data and that can quantify both the connections between clusters and the density of emitters within clusters. SuperStructure introduces for the first time the concept of connectivity between clusters, which is different from a random distribution of points at high density. In this concept, connection points are preferentially found in between clusters and this feature manifests itself in SuperStructure curves behaving as single exponentials rather than Poissonian. Because SuperStructure is parameter-free, it does not require any prior knowledge of the sample and it thus takes a crucial step toward a more standardized, portable, and democratic quantification of complex patterns and super-structures in SMLM data.

Here, we have tested the capabilities of SuperStructure first on simulated datasets, where we observed that it could capture not only the degree of connectivity between clusters but also the strength of the connections, and then on biological dSTORM data from nuclear proteins and membrane lipids. SuperStructure allowed us to discover that the speckles formed by the splicing factor SC35 are made of connected clusters. Further, that the density of emitters in those clusters is high and the connectivity between clusters even higher than that of hnRNP proteins. We argue that this may reflect the RNA-binding feature that characterizes both hnRNPs and SC35 and that may be driving the formation of interconnected nuclear super-structures. We highlight that this discovery could not be made simply by looking at clustering with traditional algorithms, as both proteins display clusters of similar size at small/intermediate ε.

We further stress that SuperStructure is perfectly suited to compare different datasets without a priori assumptions (albeit, as discussed before, segmentation to ROIs is recommended for strongly heterogeneous structures). The datasets of nuclear proteins we chose to analyze are an example of this. SAF-A, hnRNP-C, and SC-35 are three nuclear proteins involved in the metabolism of RNA at different stages, and they display three different connectivity phenotypes, which point to three different nuclear functions. In particular, SAF-A, which also plays a major role in maintaining the chromatin active loci in a decompacted state, is detected as a fully connected mesh. This finding is in agreement with a previous study that hypothesized the formation of a dynamic and RNA-interacting nuclear mesh made by SAF-A (Nozawa et al., 2017). We thus argue that SuperStructure is a useful tool for studying the structural and functional properties of this nuclear mesh. For instance, we expect that in absence of RNA, the SAF-A mesh would be disrupted and its connectivity strongly weakened (not necessarily affecting the protein clusters, which may be formed via an RNA-independent mechanism, such as phase separation by weak unspecific interactions of SAF-A’s intrinsically disordered domain). In turn, the application of SuperStructure would in this case be indispensable for understanding the link between the spatial arrangement, mechanics, and function of this nuclear protein. A similar example is given by the V(D)J locus, whereby interacting segments appear to be trapped by a protein or chromatin network whose (super-)structure is still poorly understood (Khanna et al., 2019). We argue that SuperStructure can shed light also on this problem.

In addition to all this, super-resolved chromatin tracing (Boettiger et al., 2016; Bintu et al., 2018) and super-resolved imaging of the accessible genome (Xie et al., 2020) generate complex datasets that will benefit from “beyond-traditional-clustering” algorithms. Connections between nanodomains and chromatin paths do not resemble the structure of isolated clusters but rather that of a mesh of clusters, which would be perfectly suited for quantification via the SuperStructure algorithm.

The use of SuperStructure is not limited to biological applications, and we propose it can be used as a standardized and parameter-free tool for assessing imaging technical aspects (van de Linde and Sauer, 2014; Hennig et al., 2015). One of the main issues in SMLM data, especially in dSTORM, is the evaluation of fluorophore blinking quality, as it strongly affects the localization accuracy in the analysis process. For example, an elevated blinking frequency would result in a high emitter density (per frame) and therefore in a high localization inaccuracy due to overlapping emissions. A similar detrimental effect could also be due to a poor blinking signal (few emitted photons per blinking event). As a consequence, lower localization precision of emitters may create pseudo-clusters, as well as pseudo-connections. We envisage that SuperStructure would be well suited to evaluate the blinking quality of fluorophores, for instance by measuring the emerging pseudo-connectivity in a controlled setup, such as fluorophores attached to a grid.

As discussed above, SuperStructure has been developed with the aim of going beyond “simple clustering” and in particular to measure connectivity between clusters. However, our method might be used in combination with other pairwise distance and clustering methods. For instance, one can compute Ripley’s (pairwise distance) functions to preliminarily detect if localizations are uniform or clustered and, in case, what is the average cluster radius. Yet, Ripley’s functions cannot identify single clusters or more complex structures. Thus, one could use SuperStructure to determine whether the system under investigation displays connected or isolated clusters. At the same time, by computing SuperStructure curves, one can have a firm ground to decide the value of ε that can be used as input in DBSCAN for cluster analysis. This second approach can be used, for example, to measure the size or shape of local super-structures. Indeed, one can fix ε at the value that identifies super-structures, perform a cluster analysis, and calculate the gyration tensor of the identified clusters.

**Figure S5.**
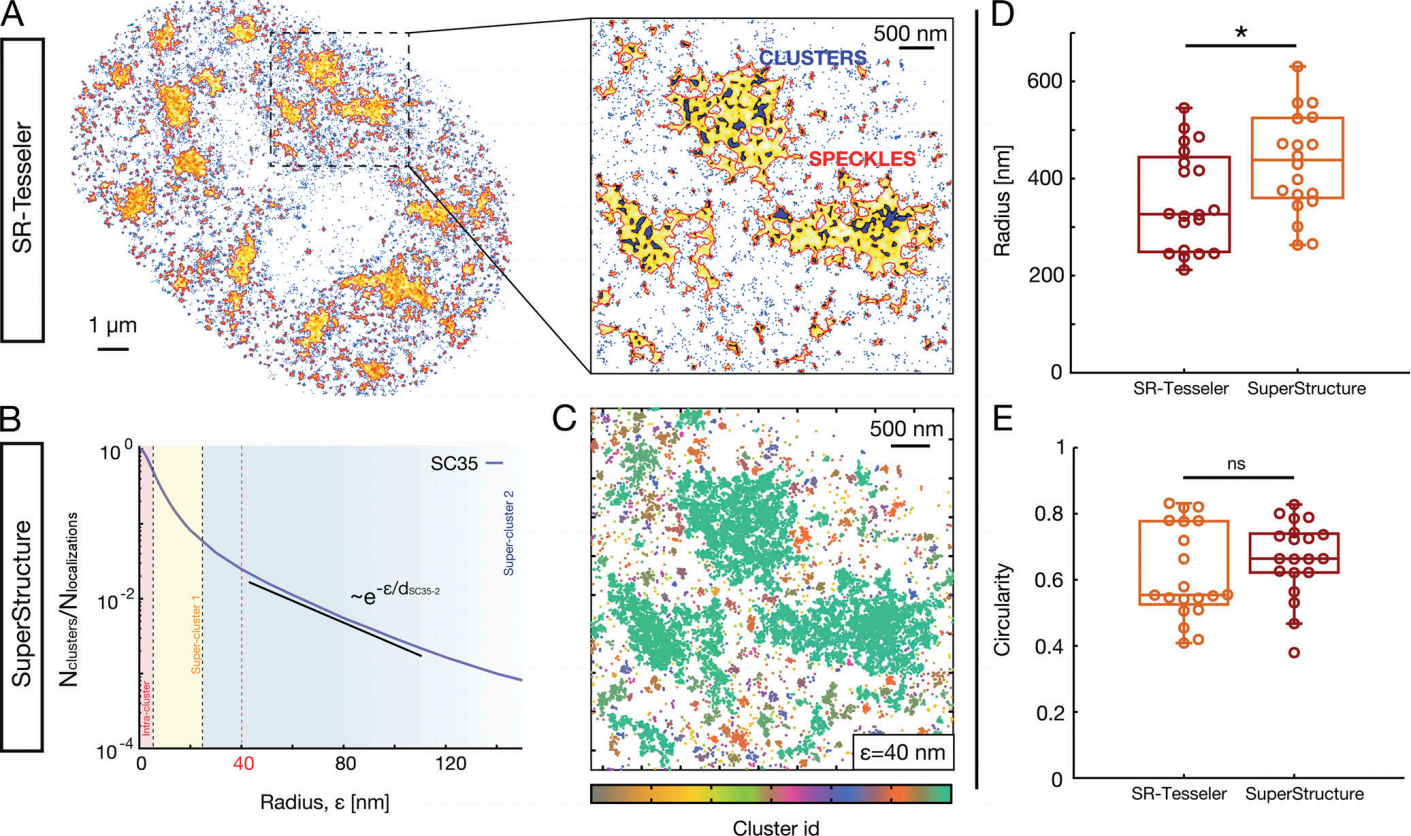
**Size and shape estimation of local super-structures emerging in SC35 dSTORM data (i.e., nuclear speckles) by using both SuperStructure and SR-Tesseler.** Analysis was performed on a single cell as proof of concept. **(A)** Super-structure detection using SR-Tesseler software, a segmentation framework based on Voronoï tessellation (constructed from the localization coordinates). Adjustments of the density factor allows the detection of structures at different density levels, such as clusters (violet) or speckles (yellow). Blue dots represent no-segmented localizations. The software was downloaded from https://github.com/flevet/SR-Tesseler/releases/tag/v1.0 and run on a Windows operating system. **(B)** SuperStructure curve of the same data. Analysis of decay regimes allows the identification of ε=40  nm as a suitable value for super-structure identifications. **(C)** Identified clusters at ε=40  nm with SuperStructure. Speckle detections are visually compatible with those of SR-Tesseler. **(D and E)** Radius and circularity of super-structures using both SR-Tesseler and SuperStructure. Both radius and circularity are very similar, showing the power of SuperStructure in computing shape and size properties. In the analysis, we considered the 20 largest identified structures (i.e., speckles). For SuperStructure, the 2D symmetric gyration tensor $\vec{R^{2}}$ was computed and diagonalized for identified super-structures. The gyration tensor components $R_{xy}^{2}\;$ are defined as $R_{xy}^{2}=\frac{1}{2N^{2}}\overset{N}{\underset{i=1}{\sum }}\overset{N}{\underset{j=1}{\sum }}(x_{i}-x_{j})(y_{i}-y_{j}),$ where N is the total number of localizations in a superstructure, and $x_{i}$ and $y_{i}$ are the x and y positions of the localization i. The diagonalization is necessary to obtain the square of the major and minor semi-axis of the speckles, namely $\gamma_{1}$ and $\gamma_{2}.$ We then calculated the speckle radius $R_{g}=\sqrt{\gamma_{1}+\gamma_{2}}$ and their circularity $c=\sqrt{\frac{\left|\gamma_{1}-\gamma_{2}\right|}{\gamma_{1}+\gamma_{2}}}.\;$ For SR-Tessler, radius and circularity parameters were obtained as output after Voronoï tessellation. P values were calculated using a Student’s *t* test: ns, P > 0.05; *, P < 0.05.

We tested the segmentation capabilities of the latter approach by estimating the radius and circularity of SC35 speckles; we observed that it yields similar results as the well-known SR-Tesseler software (Levet et al., 2015; see Fig. S5). Although SuperStructure lacks a graphical user interface, it has several advantages. First, it can be run on any operating system and can be easily automatized to run on a large number of cells. Second, since it is based on DBSCAN, the algorithm scales as $n_{\varepsilon }N^{2}$ in its simplest implementation (where $n_{\varepsilon }$ is the number of ε values used in the analysis and N is the total number of localizations). The calculations on different ε are independent, so SuperStructure scales extremely well with the number of central processing units available. For instance, the analysis of $n_{\varepsilon }=100$ values and $10^{5}$ localizations can be done on a six-core machine in ∼19 min. Third, since our algorithm is aimed at extracting beyond-simple-clustering information, it is flexible and intended to be used in combination with other pair-correlation or segmentation methods that are extensively employed for single-clustering analysis.

We conclude by highlighting that SuperStructure provides an unbiased and parameter-free estimation of (1) the density of localizations within single clusters and (2) the formation of super-structures made of connected clusters. Here, we tested SuperStructure both in simulated and cell-based SMLM datasets. Importantly, we revealed previously undocumented system-spanning structures made of connected clusters of nuclear proteins that we argue may have a functional role in shaping genome organization. The use of SuperStructure on cells under different conditions or with protein mutations is thus an exciting direction to uncover the biological significance of these newly discovered nuclear structures.

## Materials and methods

### SuperStructure algorithm

SuperStructure is an algorithm that detects and quantifies super-structures formed by interconnected clusters on SMLM datasets. Additionally, it can also evaluate the density of emitters inside clusters.

SuperStructure is mainly based on DBSCAN, a density-based algorithm to detect clusters of points in arbitrary dimensional space. The key concept underlying DBSCAN scheme is that it groups together points at high density, while it marks as outliers points in low-density regions. After defining a neighborhood size ε, a point x can be part of a cluster if the number of points N(ε,x) within a circular region Ω(ε,x) of size ε centered in x, exceeds some threshold $N_{min}\;$ (or is within the region Ω(ε,y) of another point y satisfying this condition).

The concept of clusters is subject to the choice of ε and $N_{min}$ and therefore to some sort of likeness or proximity. Furthermore, the change in number of clusters detected by DBSCAN when varying ε contains some information of the underlying distribution of points that has been overlooked.

SuperStructure progressively runs DBSCAN to detect the number of clusters $N_{c}$ within a broad range of the neighborhood parameter ε, while $N_{min}$ is kept fixed. The resulting $N_{c}\left(\varepsilon \right)$ curves, and in particular the change $dN_{c}\left(\varepsilon ,\;N_{min}\right)$ due to a small change in neighborhood parameter dε, contain fundamental information about the formation and organization of super-structures and connected clusters.

As we aim for a parameter-free algorithm, without losing generality, we fix $N_{min}=0,$ which means no minimum number of other emitters necessary in the neighborhood to define a localization as part of a cluster. For ε=0, any point is found to be a cluster by itself. Then, points merge upon increasing ε→  ε + dε, resulting in $dN_{c}/d\varepsilon \leq \;0\;\forall \;\varepsilon .$ Additionally, the larger $\left|dN_{c}/d\varepsilon \right|,$ the more identified clusters are coalescing together for a certain ε.

At  ε smaller than the typical (true, rather than the one detected by DBSCAN) cluster size, the decay of $dN_{c}/d\varepsilon$ is determined by the intra-cluster density of points $\rho_{em}$ (intra-cluster regime), as they are the points at the highest density. The decay of this regime is Gaussian and it is described by the Poisson function:

$$N_{c}\left(\varepsilon \right)=\overset{m}{\underset{k=0}{\sum }}c_{k}\frac{{\left(\pi \rho_{em}\varepsilon^{2}\right)}^{k}}{k!}\;e^{-\pi \rho_{em}\varepsilon^{2}}.$$ (1)

To understand the origin of this functional form, let us imagine to apply the SuperStructure algorithm by setting $N_{min}=0$ and increasing the radius ε. For sufficiently small ε, every point is considered as a single cluster itself, as no other points are detected in its neighborhood. However, by increasing ε, the probability of finding another point in the neighborhood increases, implying that points start to merge in bigger clusters for small ε. It is then legitimate to argue that the number of detected clusters $N_{c}$ decreases (with ε) as the probability of not finding any other emitter in the neighborhood. This is the so-called Poisson avoidance function $N_{c}\left(\varepsilon \right)=P\left(n\left(\varepsilon \right)=0\right)=e^{-\pi \rho_{em}\varepsilon^{2}},$ and it is a good approximation for very small ε, where the contribution of clusters formed by two emitters dominates over clusters formed by three or more points. For larger ε, this function underestimates the number of detected clusters. The number of detected clusters can therefore be described by the probability of not finding more than m particles in the circle of radius ε. The function we are seeking is the linear combination of the probabilities of not finding any other point in the neighborhood and finding one or more other points (up to m−1). Being the probability of finding k particles $P\left(n\left(\varepsilon \right)=k\right)=\frac{{\left(\pi \;\rho_{em}\;\varepsilon^{2}\right)}^{k}}{k!}e^{-\pi \rho_{em}\varepsilon^{2}},$ it is then straightforward to get the functional form of Eq. 1.

Note that $c_{k}=1/(k+1)$ in Eq. 1 is to avoid overcounting clusters. In fact, if we consider two points within distance ε from each other (and hence in the same cluster), both points will count toward P(n(ε)=1), so this contribution must be divided by 2, etc. Importantly, Eq. 1 displays a natural length-scale $\kappa_{0}={\left(\pi \;\rho_{em}\right)}^{-1/2}$ that is intrinsically determined by the internal density of emitters $\rho_{em}.$ Therefore, $\rho_{em}$ is a parameter that can be quantified by fitting the $N_{c}\left(\varepsilon \right)$ curve, and it can also be used to quantify the approximate upper limit of this regime (with 99% confidence level):

$$\varepsilon^{*}≃\;3\kappa_{0}\;=\;3/\sqrt{\pi \;\rho_{em}}=\;3R_{cl}/\sqrt{N_{em}},$$ (2)

where $R_{cl}$ is the average cluster radius and $N_{em}$ is the average number of localizations within a single cluster. We successfully tested that SuperStructure curves are well fitted by Eq.1 up to m=2 using a system where we simulated localization of points inside a single cluster (see Fig. S1).

At ε of the order than the typical (true) cluster size, the decay is determined by the rate at which distinct clusters merge upon ε→ε + dε (first super-cluster regime). This merging can be due to either (1) distinct clusters starting to overlap as their distance is smaller than ε or (2) the presence of points, which we call connections, bridging two clusters. In case of total absence of connectivity and a homogeneous cluster distribution, the merging is only due to the random positioning of clusters, and therefore, it also follows a Poisson function:

$$N_{c}\left(\varepsilon \right)\;=f\;\overset{m}{\underset{k=0}{\sum }}c_{k}\frac{{\left(\pi \;\rho_{cl}\varepsilon^{2}\right)}^{k}}{k!}e^{-\pi \rho_{cl}\varepsilon^{2}},\;\;$$ (3)

where f is a normalization factor and $\rho_{cl}$ the density of clusters. We observed that SuperStructure curves of simulated systems are well fitted by using m=1. This equation holds also in presence of noise, but in that case, $\rho_{cl}\to \rho_{cl}+\rho_{noise}$ (see Fig. S2). The decay is different in presence of connections between clusters; connected clusters will merge at smaller ε than unconnected ones (assuming same distance between the centers of clusters). In particular, the larger the number of connections or of the local density of connection points $\rho_{conn}$ (i.e., thicker connections), the faster the merging of bridged clusters as a function of ε and thus the larger $\left|dN_{c}/d\varepsilon \right|.$ The functional form of this second regime is exponential in presence of connections:

$$N_{c}\left(\varepsilon \right)=g\cdot e^{-\varepsilon /\lambda },\;$$ (4)

where g is a normalization factor and λ the decay length quantifying the rate of decay and therefore the connectivity. This decay length can be used to discern systems that exhibit either different grades of connectivity or homogeneous meshes at different densities. Note λ purely quantifies the connectivity only when the cluster density $\rho_{cl}$ is small and homogeneous, as we could have underlying highly dense clusters overlapping and therefore merging. We showed that $\lambda ∼\rho_{cl}^{-1/2}$ and therefore the pure connectivity decay length can be further evaluated if the density of clusters is known: $\lambda^{*}∼\lambda /\rho_{cl}^{-1/2}.$

We need to stress that by choosing $N_{min}=0,$ connections will also be considered as points to be merged. However, it is important that we identify connection points as having a lower local density $\rho_{conn}$ than the groups of points that are bridged by them (clusters). In this way, they will merge in this second regime to form super-structures. The limiting case in which the local density of connection points is the same as the one in the clusters at the two ends of the connections is indistinguishable from the case of one elongated cluster. A special case is that in which both clusters and connections have the same density of points but the connections are slightly detached from the clusters, thus forming three independent clusters at intermediate ε, which may then merge (we assume this to be a rare event). The above reasoning can be extended to multiply connected clusters via the analysis of pairwise connections.

At larger ε, we could have additional super-cluster regimes if the system is heterogeneous. Most common cases showing two (or more) super-cluster regimes are the following: (1) inhomogeneous system displaying different connectivities at different length-scales, (2) connected clusters embedded in a noisy environment (in this case we observe an exponential followed by a Poissonian decay), and (3) unconnected clusters within a random noise and/or unconnected clusters at different densities (in this case, we observe two or more Poissonian decays).

### SuperStructure pipeline

To apply SuperStructure, we adopt the following steps.

(1) Generation of SuperStructure curves.**We run SuperStructure on a SMLM dataset by first masking our data in the ROI, such as the nucleus for nuclear proteins as mentioned in the section below. Then, we choose a ε range to analyze. For example, in SMLM datasets of nuclear proteins a typical choice is ε∈[0:200] nm with dε=2 nm. One should notice that lower dε may be necessary for fitting the intra-cluster regime. SuperStructure curves are generated by progressively running DBSCAN clustering algorithm on the SMLM dataset in the chosen ε range (and $N_{min}=0).$ The DBSCAN software we use is from https://github.com/gyaikhom/dbscan, and the progressive run is performed with bash scripts available in the repository. SuperStructure output curves are saved in a three-column file $(\varepsilon ,\;N_{cl},\;N_{cl}/N_{loc}),$ where $N_{cl}$ is the number of detected clusters for the corresponding ε and $N_{loc}$ the number of total localizations. Additionally, the classification of localizations in clusters is saved on a separate file for every ε.

(2) Evaluation of SuperStructure regimes. As a second step, we evaluate regimes by plotting and investigating SuperStructure curves (we adopt a log scale in the y axis). This step includes a preliminary check for the number of regimes and their decay behavior (exponential versus Poissonian). In the case we observe a single Poissonian behavior, we can state that the dataset does not show any, or very limited, connectivity, and therefore, we are in presence of homogeneous isolated clusters (and eventually noise). Limited connectivity needs to be checked with a cluster analysis and direct dataset observation in case noise has obscured an exponential decay. On the other hand, if we observe a single exponential regime (a straight line in a log-linear plot), we conclude that the system is made of fully connected clusters. If SuperStructure curves show multiple super-cluster regimes, it is likely that the system is heterogeneous. Indeed, multiple exponential regimes may reflect heterogeneous/multiscale connectivities combined with heterogeneous distributions of clusters. Alternatively, we may find also a combination of exponential and Poissonian regimes, and in this case, the system may be made of connected clusters embedded in a noisy region. Other more complex combinations may be possible; however, one should notice that in heterogeneous systems, it might be difficult to recognize and fit super-cluster regimes. To clarify these contributions, it is useful to combine the analysis of SuperStructure curves with a direct observation of the dataset and identified structures and to run SuperStructure on smaller ROIs to analyze different regions of the sample with similar structural phenotypes. Nonetheless, SuperStructure will be able to unambiguously detect differences in connectivity and behaviors in, for example, samples that have been subjected to different conditions or expressing mutated proteins.

(3) Fit of SuperStructure regimes. Once regimes have been identified, one needs to define the boundaries where regimes crossover from one to another. This can be either done manually or by using a preemptive goodness-of-fit test (this procedure would also define fitting ranges). The intra-cluster regime is typically fitted with a Poisson equation (Eq. 1) to evaluate the density of emitters inside clusters as well as obtain an estimation of the upper limit of the intra-cluster regime (using Eq. 2). For super-cluster regimes, we use Eq. 3 if they show a Poissonian decay (curved on a log-linear plot) or Eq. 4 if they otherwise appear straight on a log-linear plot; from the latter, we quantify the connectivity parameter λ. We can then additionally calculate the cluster density $\rho_{cl}$ to extract the pure connectivity part $\lambda^{*}=\lambda /{\rho_{cl}}^{-1/2}.$ The cluster density $\rho_{cl}$ can be computed by performing a cluster analysis with DBSCAN on local circular regions representative of that decay regime and by fixing ε at the start of that regime (e.g., by counting the number of clusters one obtains by fixing ε at the beginning of the yellow area in Fig. 3). In the section below and in Fig. S3, we describe in detail the procedure for λ normalization for the nuclear protein datasets. Finally, and optionally, it is also possible to define a single function fitting the entire curve by either (1) defining a piecewise function where every piece is the fit of the corresponding regime or (2) adding together the contribution of the different regimes (appropriately weighted). We performed fits with a combination of bash and gnuplot scripts available in the repository.

### Simulated dataset generation and SuperStructure analysis

The simulated dataset consists of spatially homogeneous and interconnected clusters randomly distributed on a plane. We set to work with clusters made by taking random clusters centers on the plane and by sampling $N_{em}=80$ emitters within a Gaussian of standard deviation $\sigma_{em}=\;20\;nm,$ thereby setting the cluster radius to $R_{cl}=2\;\sigma_{em}=\;40\;nm$ with a 95% confidence and the intra-cluster emitters density at $\rho_{em}=16,000\;\mu m^{-2}.$ The clusters are positioned in a L=3.5 μm large area, and their number $N_{cl}$ is varied in order to consider different clusters densities. In the example shown in the main text, we fixed $N_{cl}=100,$ thus fixing a cluster density to approximately $\rho_{cl}\;=8.2\;\mu m^{-2},$ roughly similar to the values found in experiments for some nuclear proteins. Pairs of clusters are connected with probability $p_{r}$ if they are positioned closer than a distance b=1 μm. The value of $p_{r}$ is calculated as the ratio between the actual drawn connections and $N_{cl}\left(N_{cl}-1\right)/2,$ which is the maximum possible connections (i.e., when every cluster is connected with every other cluster). To generate a single connection, we considered the vector joining the centers of two clusters and sampled one emitter with probability $p_{r}{}_{conn}$ every 10 nm. Emitters are sampled from a 2D Gaussian centered on the vector connecting the two clusters centers and with a width $\sigma_{conn}=10\;nm.$ In the main text, we fixed $p_{r}{}_{conn}=0.5.$ Note that $p_{r}$ controls the number of connections, while $p_{r}{}_{conn}$ controls their density, $\rho_{conn}.$ We generated at least 20 independent replicas for each simulated dataset using a combination of bash and python scripts, and then we ran SuperStructure analysis in the range ε∈[0:400] nm with a change dε=2 nm. If not differently specified, the first super-cluster regime was fitted with Eq. 4 for ε∈[15:60], while the second super-cluster regime was fitted with either Eq. 3 (unconnected systems) or Eq. 4 (connected systems) for ε∈[70:300].

### Cell preparation for dSTORM imaging of nuclear proteins

#### 

hTERT-RPE1 cells (catalog no. ATCC-CRL-4000; American Type Culture Collection) were grown overnight in an eight-well Lab-Tek II Chambered Coverglass–1.5 borosilicate glass (Thermo Fisher Scientific) at 37°C at initial concentration of $10^{5}\;cells/ml\;$ in 400 μl (∼40% confluency). We fixed the cells with 4% PFA (Sigma-Aldrich) for 10 min, washed three times in PBS, permeabilized with 0.2% Triton X-100 (Sigma-Aldrich) for 10 min, washed three times in PBS, and blocked with 1% BSA (Sigma-Aldrich) for 10 min.

Immunofluorescence labeling was done by exposing the cells for 2 h to (1) hnRNP-U polyclonal rabbit antibody (A300-690A; Bethyl Laboratories) at 10 μg/ml, (2) hnRNP-C1/C2 (4F4) mouse monoclonal antibody (sc-32308; Santa Cruz Biotechnology) at 0.2 μg/ml, or (3) SC-35 mouse monoclonal antibody (ab11826; Abcam) at 2 μg/ml and then three washes. Cells were then exposed for 1 h to secondary antibody. The secondary antibody was made by AffiniPure $F{\left(ab'\right)}_{2}$ fragment donkey anti-rabbit or donkey anti-mouse IgG (H+L; 711–006-152 and 715–007-003, Jackson ImmunoResearch Europe Ltd.) conjugated to the organic fluorophore CF647 (92238A-IVL; Sigma-Aldrich) at a stochiometric ratio of ∼1. After that, cells were washed three times in PBS.

Oxygen scavenger imaging buffer based on the glucose oxidase enzymatic system (GLOX) for dSTORM was prepared fresh. The recipe employed was similar to that used previously (McSwiggen et al., 2019). We mixed (1) 5.3 ml of 200 mM Tris and 50 mM NaCl solution with (2) 2 ml of 40% glucose solution, (3) 200 μl GLOX, (4) 1.32 ml of 1 M 2-mercaptoethanol (Sigma-Aldrich), and (5) 100 μl of 50 μg/ml DAPI solution (Sigma-Aldrich). The GLOX solution was made by mixing 160 μl of 200 mM Tris and 50 mM NaCl with 40 μl catalase from bovine liver (Sigma-Aldrich) and 18 mg glucose oxidase (Sigma-Aldrich).

The 8.9-ml final solution was enough to fill the chambers of the eight-well dish; a coverglass was sealed at the top of the dish to prevent inflow of oxygen.

### dSTORM acquisition of nuclear proteins

We performed 3D-STORM acquisitions using a Nikon N-STORM total internal reflection fluorescence system (TIRF) with Eclipse Ti-E inverted microscope and laser TIRFilluminator (Nikon). We equipped the microscope with a CFI SR HP Apo TIRF 100× objective lens (N.A. 1.49) and applied a 1.5× additional optical zoom. We also used a cylindrical astigmatic lens to obtain elliptical shapes for emitters that reflect their z-position (Huang et al., 2008). Laser light was provided via a Nikon LU-NV laser bed with 405-, 488-, 561-, and 640-nm laser lines. In particular, CF647 fluorophores were stochastically excited using the 640-nm laser beam with an additional 405-nm weak pulse. Images were acquired with an Andor iXon 897 EMCCD camera (Andor Technologies). The z-position was stabilized during the entire acquisition by the integrated perfect focus system. Acquisition were performed at room temperature.

For every nucleus, we acquired a stack of 20,000 frames at 19-ms exposure time by using the Nikon NIS-Element software. Acquired images have a 256 × 256 pixel resolution with pixel size equal to 106 nm. For every condition (SAF-A, hnRNP-C, and SC35), we acquired six nuclei (i.e., six independent datasets).

### Raw images and post-processing analysis for nuclear protein data

The raw stack of frames was initially segmented based on a DAPI marker to carefully mask out the extranuclear signal. Then, frames were analyzed using FIJI (Schindelin et al., 2012) and in particular the Thunderstorm plugin (Ovesný et al., 2014). First, we filtered them by using Wavelet functions to separate signal from noise. The B-Spline order was set to 3 and the B-Spline scale to 2.0 as suggested previously (Ovesný et al., 2014) for localizations of ∼5 pixels. To localize the emitters centroids, we thresholded filtered images (threshold value was set 1.2 times the standard deviation of the first Wavelet function) and calculated the local maximum relative to the eight nearest neighbors. Finally, we fitted the emitters signal distribution with elliptical gaussians (ellipses are necessary for z-position reconstruction) using the weighted least-square method and by setting 3 pixels as fitting radius and 1.6 pixels as initial sigma.

Localized data were then postprocessed using the same plugin. We corrected the xy drift using a pair-correlation analysis, filtered data with a position uncertainty < 40  nm,  restricted the z-position to the interval [−100:100]  nm, and projected the data in a 2D plane, as the z-axis precision is ∼100 nm.

Reconstructed images shown in the main text were created by using the average shifted histograms method of the same plugin with 10× magnification (final resolution set to 10.6 nm/pixel).

### SuperStructure analysis for nuclear protein data

SuperStructure analysis was run on the entire nuclear region by setting $N_{min}=0$ and by increasing ε in the range [0:200] nm, and “all-nucleus” curves were generated for six independent nuclei. We set the change rate dε=0.25 nm for ε∈[0:10] nm and dε=10  nm for ε∈[10:200] nm. This choice was due to the higher resolution necessary to extract intra-cluster information at small ε. As shown in Fig. 3, SuperStructure all-nucleus curves show that SAF-A has a single exponential super-cluster regime, while hnRNP-C and SC35 have two regimes. In the case of hnRNP-C, the second regime is due to weakly connected and sparse clusters in nucleoli, while in SC35 it is due to the cluster/connectivity heterogeneity in the system (i.e., speckles). Therefore, we additionally run SuperStructure analysis on local ROIs for hnRNP-C and SC35 to obtain the isolated contribution for the first super-cluster regime. In particular, for hnRNP-C, we considered five independent circular ROIs per nucleus with radius r=1.5 μm within the nuclear mesh; for SC35, we considered five independent circular ROIs per nucleus with radius r=0.5 μm within speckles. We ran the analysis on these ROIs and generated SuperStructure “local” curves (five for each nucleus).

The values of the intra-cluster density $\rho_{em}$ were extracted by fitting with Eq. 1 the intra-cluster regime in the all-nucleus curves in the range ε∈[0,3] nm. Resulting average values are $\rho_{em}^{hnRNP-C}=7,973\pm 1,732\;\mu m^{-2},$ $\rho_{em}^{SAF-A}=16,998\;\pm \;2,444\;\mu m^{-2},$ and $\rho_{em}^{SC35}=18,680\;\pm \;1,520\;\mu m^{-2}.$

Then, we identified the super-cluster regimes of interest: the first super-cluster regimes of SAF-A and hnRNP-C and both super-cluster regimes of SC35 (SC35-1 and SC35-2). For SAF-A and SC35-2, the decay length λ was obtained by fitting all-nucleus curves with Eq. 4. For hnRNP-C and SC35-1, we fitted the local curves (five curves per nucleus) and then averaged λ values obtained from different local curves in the same nucleus. Fit ranges are ε∈[16,100] nm for SAF-A, ε∈[14,70] nm for hnRNP-C, ε∈[8,20] nm for SC35-1, and ε∈[40,150] nm for SC35-2.

Finally, the values of λ for SAF-A, hnRNP-C, SC35-1, and SC35-2 were normalized by the cluster density: $\lambda^{*}=\lambda \;/\rho_{cl}^{-1/2}.$ In the case of SAF-A and SC35-2, the normalization was performed for λ for every nucleus by using the average cluster density $\rho_{cl}\;$ of that nucleus. In particular, $\rho_{cl}\;$ was calculated as the average of the cluster density in five independent circular regions of radius r in the same nucleus as shown in the example of Fig. S3 A. In the case of hnRNP-C and SC35-1, where λ values were obtained from local curves, the normalization of λ was performed using the cluster density of the same local region; then, $\lambda^{*}$ values obtained from different regions in the same nucleus were averaged (see Table S1). The number of clusters estimation (to calculate the cluster density) was made with DBSCAN by setting $N_{min}=0$ and ε close to the beginning of the exponential regime of interest, as shown in Fig. S3 B, and by keeping only clusters with at least 30 particles. To compute the cluster density, for SAF-A and hnRNP-C, we set local circular regions of radius r=1.5 μm and fixed ε=20  nm for cluster analysis (for hnRNP-C, we used the same local regions as defined above). For SC35, we considered two sets of local regions: (1) inside speckles to normalize the shorter decay length, where we used ROIs with radius r=500  nm and fixed ε=10  nm for cluster analysis (same regions as above); and (2) outside speckles to normalize the longer decay length, where we used ROIs with radius r=1.5 μm and ε=40  nm for cluster analysis. Average nuclear values of λ, $\rho_{cl}\;,$ and $\lambda^{*}$ are shown in Table S1.

### SuperStructure analysis of ceramide data

SuperStructure analysis was run on the two ceramide datasets provided by the authors from Burgert et al. (2017), namely +bSMase and −bSMase, by setting $N_{min}=0$ and ε∈[0:200]. We set dε=0.5 nm for ε∈[0:10] nm and dε=2  nm for ε∈[10:200] nm. This choice was due to the higher resolution necessary to extract intra-cluster information at small ε. From the curves in Fig. 4 B, it is clear that there is no strong connectivity (we observe a Poissonian decay). Therefore, we identified free unclustered emitters as noise. We additionally ran SuperStructure in 16 independent local circular regions of radius r=1.5 μm to extract the quantities of interest. In particular, we measured the average densities of total localizations, $\rho_{loc}^{+}=595\pm 130\;\mu m^{-2}$ and $\rho_{loc}^{-}=475\pm 87\;\mu m^{-2},$ respectively, for +bSMase and −bSMase treatment. This is in accordance with results in the original paper. Then, we fitted local SuperStructure curves in the intra-cluster regime with Eq. 1 for ε∈[0:3] nm: $\rho_{em}^{+}=22,391\pm 3,306\;\mu m^{-2}$ and $\rho_{em}^{-}=15,505\pm 3,470\;\mu m^{-2},$ respectively, for +bSMase and −bSMase treatments. Finally, we fitted local SuperStructure curves in the super-cluster regime with Eq. 3 in the range ε∈[50:200] nm for +bSMase and ε∈[60:200] nm for −bSMase (the difference in fit starting value is explained by a horizontal shift between the two curves): $\rho_{sc}^{+}=62.01\;\pm \;20.76\;\mu m^{-2}$ and $\rho_{sc}^{-}=43.56\pm 11.05\;\mu m^{-2}.$ These two values are in accordance with the sum of cluster density and noise at the ε value were the fit starts. We additionally performed a cluster analysis with DBSCAN, and results are in agreement with the original paper (see Fig. S4 for details). To verify that there is no limited connectivity hidden by noise, we performed a cluster analysis at two different values of ε and monitored the change in density of clusters and density of free emitters (see Fig. S4 for details).

### Data availability

The simulated and experimental datasets that support the findings of this study are available from the corresponding authors upon request.

### Code availability

The code for the generation of SuperStructure curves is available at https://git.ecdf.ed.ac.uk/dmichiel/superstructure.

### Online supplemental material

Fig. S1 shows a simulated distribution of points inside a single cluster and how it is well represented by Eq. 1 in Materials and methods. Fig. S2 shows SuperStructure curves for simulated datasets of connected clusters in different conditions, including systems with different cluster densities, systems embedded in a noisy environment, and fully connected meshes. Fig. S3****shows how the normalization of λ was performed in nuclear protein data (exhaustively explained in Materials and methods) and that nuclear proteins connectivity is not a technical artifact. Fig. S4 shows that there is no local connectivity in ceramide data and confirms the original paper’s results on ceramide cluster size. Fig. S5 shows SuperStructure + DBSCAN segmentation capabilities by estimating the radius and circularity of SC35 speckles alongside SR-Tesseler software.****Table S1 recapitulates values for λ, $\rho_{cl},$ and $\lambda^{*}$ in nuclear protein data.

## Supplementary Material

Table S1. Decay length λ, detected cluster density ρ_cl_, and normalized decay length λ* = λ / ρ_cl_^−1/2^ for SAF-A, hnRNP-C, and SC-35 (in both super-cluster regimes, SC35-1 and SC35-2)
