# Supplementary material for: Parameter-free molecular super-structures quantification in single-molecule localization microscopy
Source: J Cell Biol. 2021 Mar 18;220(5):e202010003. doi: 10.1083/jcb.202010003 (PMC7980255; doi:10.1083/jcb.202010003)
Supplement: Table S1. — Decay length λ, detected cluster density ρcl, and normalized decay length λ* = λ / ρcl−1/2 for SAF-A, hnRNP-C, and SC-35 (in both super-cluster regimes, SC35-1 and SC35-2) [file JCB_202010003_TableS1.docx]

**Supplemental Table**

| **SAF-A** | | | |
| --- | --- | --- | --- |
| ***Nucleus*** | $\boldsymbol{\lambda}\left( \boldsymbol{nm} \right)$ | $\boldsymbol{\rho}_{\boldsymbol{cl}}\left( \boldsymbol{\mu}\boldsymbol{m}^{\boldsymbol{-2}} \right)$ | $\boldsymbol{\lambda}^{\boldsymbol{*}}\boldsymbol{/}\boldsymbol{10}^{\boldsymbol{-2}}$ |
| 1 | 18.75 | 8.686 | 5.526 |
| 2 | 17.46 | 9.846 | 5.477 |
| 3 | 18.94 | 10.24 | 6.062 |
| 4 | 15.30 | 11.80 | 5.255 |
| 5 | 16.57 | 10.87 | 5.463 |
| 6 | 20.41 | 8.432 | 5.926 |
| Avg | 17.90 ± 1.68 | 9.978 ± 1.173 | 5.618 ± 0.282 |

| **hnRNP-C** | | | |
| --- | --- | --- | --- |
| ***Nucleus*** | $\boldsymbol{\lambda}\left( \boldsymbol{nm} \right)$ | $\boldsymbol{\rho}_{\boldsymbol{cl}}\left( \boldsymbol{\mu}\boldsymbol{m}^{\boldsymbol{-2}} \right)$ | $\boldsymbol{\lambda}^{\boldsymbol{*}}\boldsymbol{/}\boldsymbol{10}^{\boldsymbol{-2}}$ |
| 1 | 11.82 | 8.912 | 3.520 |
| 2 | 12.93 | 6.621 | 3.320 |
| 3 | 10.07 | 12.13 | 3.492 |
| 4 | 9.463 | 12.27 | 3.374 |
| 5 | 8.920 | 13.27 | 3.229 |
| 6 | 10.54 | 11.86 | 3.613 |
| Avg | 10.62 ± 1.37 | 10.92 ± 2.37 | 3.425 ± 0.129 |

| **SC35-1 (first regime)** | | | |
| --- | --- | --- | --- |
| ***Nucleus*** | $\boldsymbol{\lambda}\left( \boldsymbol{nm} \right)$ | $\boldsymbol{\rho}_{\boldsymbol{cl}}\left( \boldsymbol{\mu}\boldsymbol{m}^{\boldsymbol{-2}} \right)$ | $\boldsymbol{\lambda}^{\boldsymbol{*}}\boldsymbol{/}\boldsymbol{10}^{\boldsymbol{-2}}$ |
| 1 | 5.882 | 23.17 | 2.693 |
| 2 | 5.094 | 32.85 | 2.898 |
| 3 | 4.777 | 36.92 | 2.818 |
| 4 | 4.797 | 38.96 | 2.976 |
| 5 | 4.591 | 35.65 | 2.534 |
| 6 | 7.033 | 19.10 | 2.937 |
| Avg | 5.362 ± 0.855 | 31.11 ± 7.38 | 2.809 ± 0.154 |

| **SC35-2 (second regime)** | | | |
| --- | --- | --- | --- |
| ***Nucleus*** | $\boldsymbol{\lambda}\left( \boldsymbol{nm} \right)$ | $\boldsymbol{\rho}_{\boldsymbol{cl}}\left( \boldsymbol{\mu}\boldsymbol{m}^{\boldsymbol{-2}} \right)$ | $\boldsymbol{\lambda}^{\boldsymbol{*}}\boldsymbol{/}\boldsymbol{10}^{\boldsymbol{-2}}$ |
| 1 | 36.02 | 5.517 | 8.461 |
| 2 | 29.46 | 4.838 | 6.479 |
| 3 | 27.14 | 5.404 | 6.309 |
| 4 | 35.16 | 4.527 | 7.481 |
| 5 | 31.48 | 4.584 | 6.740 |
| 6 | 30.33 | 4.951 | 6.748 |
| Avg | 31.60 ± 3.11 | 4.970 ± 0.377 | 7.036 ± 0.735 |

**Table SI.** Decay length $\lambda$, detected clusters density $\rho_{cl}$ and normalised decay length $\lambda^{*}=\lambda/\rho_{cl}^{-1/2}$ for SAF-A, hnRNP-C and SC-35 (in both super-cluster regimes SC35-1 and SC35-2). Both single-nucleus values and average over nuclei ($\pm$ standard deviation) are shown. For SAF-A and SC35-2, $\lambda$ was obtained by fitting “all-nucleus” SuperStructure curves, i.e. curves where the entire nucleus was analysed. On the other hand, for hnRNP-C and SC35-1, $\lambda$ was obtained by fitting “local” SuperStructure curves, i.e. curves where local circular regions were analysed as explained in Methods. In the latter case, nuclear values showed in the table are the result of an average over $5$ independent “local” values within the same cell.
